# Design and Synthesis of Hepatitis B Virus (HBV) Capsid Assembly Modulators and Evaluation of Their Activity in Mammalian Cell Model

**DOI:** 10.3390/ph15070773

**Published:** 2022-06-22

**Authors:** Karina Spunde, Brigita Vigante, Unda Nelda Dubova, Anda Sipola, Irena Timofejeva, Anna Zajakina, Juris Jansons, Aiva Plotniece, Karlis Pajuste, Arkadij Sobolev, Ruslan Muhamadejev, Kristaps Jaudzems, Gunars Duburs, Tatjana Kozlovska

**Affiliations:** 1 Latvian Biomedical Research and Study Centre, LV-1067 Riga, Latvia; undadubova@gmail.com (U.N.D.); irena@biomed.lu.lv (I.T.); anna.zajakina@gmail.com (A.Z.); jansons@biomed.lu.lv (J.J.); tatyana@biomed.lu.lv (T.K.); 2 Latvian Institute of Organic Synthesis, LV-1006 Riga, Latvia; anda@osi.lv (A.S.); aiva@osi.lv (A.P.); kpajuste@osi.lv (K.P.); arkady@osi.lv (A.S.); muhamadejev@osi.lv (R.M.); kristaps.jaudzems@osi.lv (K.J.); gduburs@osi.lv (G.D.)

**Keywords:** hepatitis B virus, antivirals, full-length HBV core, capsid assembly, capsid assembly modulator, heteroaryldihydropyrimidines, Bay 41-4109

## Abstract

Capsid assembly modulators (CAMs) have emerged as a promising class of antiviral agents. We studied the effects of twenty-one newly designed and synthesized CAMs including heteroaryldihydropyrimidine compounds (HAPs), their analogs and standard compounds on hepatitis B virus (HBV) capsid assembly. Cytoplasmic expression of the HBV core (HBc) gene driven by the exogenously delivered recombinant alphavirus RNA replicon was used for high level production of the full-length HBc protein in mammalian cells. HBV capsid assembly was assessed by native agarose gel immunoblot analysis, electron microscopy and inhibition of virion secretion in HepG2.2.15 HBV producing cell line. Induced fit docking simulation was applied for modelling the structural relationships of the synthesized compounds and HBc. The most efficient were the HAP class compounds—dihydropyrimidine 5-carboxylic acid *n*-alkoxyalkyl esters, which induced the formation of incorrectly assembled capsid products and their accumulation within the cells. HBc product accumulation in the cells was not detected with the reference HAP compound Bay 41-4109, suggesting different modes of action. A significant antiviral effect and substantially reduced toxicity were revealed for two of the synthesized compounds. Two new HAP compounds revealed a significant antiviral effect and a favorable toxicity profile that allows these compounds to be considered promising leads and drug candidates for the treatment of HBV infection. The established alphavirus based HBc expression approach allows for the specific selection of capsid assembly modulators directly in the natural cell environment.

## 1. Introduction

Despite the fact that a safe and effective hepatitis B vaccine and a series of medications against hepatitis B virus (HBV) have been developed, there are >350 million chronic carriers of HBV in the world, of which nearly 1 million die annually [1,2]. Up to 40% of the cases of chronic hepatitis B (CHB) progress to liver cirrhosis, hepatic decompensation, and hepatocellular carcinoma (HCC), which is a leading cause of cancer-related morbidity and mortality worldwide [3].

The risk of developing HCC has been shown to be at least 100-fold higher in patients with chronic HBV infection than in people not infected with HBV. Moreover, there is a direct relationship between the burden of the virus and the risk of developing HCC. Consequently, effective antiviral therapy is essential for HCC prevention, as well as reducing the risk of cancer relapse and increasing survival [4]. Despite therapy with efficient inhibitors of HBV reverse transcriptase, a complete cure of CHB can rarely be achieved with this approach [4,5]. Treatment is mainly hampered by the epichromosomal viral genome—covalently closed circular DNA (cccDNA) persistence in the cell. Previous treatment methods have kept the transcription of cccDNA in the cell inactive but were not able long-term disease control [6]. Various studies have shown that up to 50% of patients undergoing long-term nucleoside/nucleotide therapy develop therapy-resistant HBV mutants [7,8,9].

The small HBV genome encodes only seven proteins, each of which has a different function in the life cycle of the virus. Due to the complexity of the virus replication cycle, the most likely effective treatment could be through a combination of medicines that target different steps in the HBV replication cycle. A promising molecular target for anti-HBV agents with a very low probability of developing resistance is the self-assembly of HBV capsids, as even slight changes in the dynamics of capsid assembly may impair the packaging and release of the viral genome [10]. In the HBV life cycle, the proper assembly of the hepatitis B core (HBc) protein is critical for HBV pregenome encapsidation, the development of infectious virions and viral cccDNA amplification in the nucleus [11].

**Figure 1:**
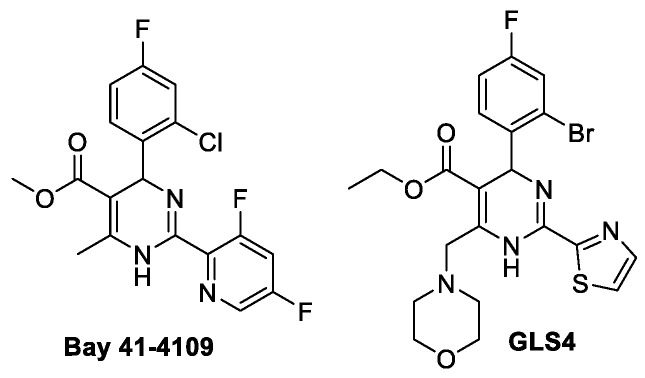
Structures of capsid assembly modulators: Bay 41-4109 and GLS4 [18].

The promising HBV chemotherapy approach is based on disabling the proper assembly of capsids. In order to function, capsids must be precisely built from the basic elements of the 120 core protein dimers to create the correct architecture. Capsids should protect the viral nucleic acids inside and outside the cell but ensure a prompt release of the viral genome upon infection. As capsid self-assembly is a virus specific process that has no analogues in human, destroying this process could represent an efficient strategy for patient treatment. To date, medicinal chemistry has been mainly working on the effects of small molecules on enzymes and receptors. Currently, as a fundamental novelty, the effects of small molecules on protein–protein interactions, agents binding to protein–protein contact surfaces (PPSs) or allosteric sites, is arising [12]. These approaches are unique—the effects relate to large protein surfaces with poorly defined binding pockets compared to the usual binding sites of biologically active substances at the ion channels, enzymes, or receptors. Modification of PPSs for viral capsids from the point of view of medicinal chemistry was until recently considered a very serious, complex, and practically hopeless task. However, significant results have been achieved in the last decade to change this view [12,13,14,15]. Several compounds of the heteroaryldihydropyrimidine (HAP) class, such as Bay 41-4109 and GLS4 [16], representatives of capsid assembly modulators (CAMs), were discovered to be capable of suppressing HBV replication by disturbing the proper self-assembly of capsids and causing aberrant capsid structure formation [17]. Bay 41-4109 and GLS4 (Figure 1) [18], according to clinical trial data, produce strong and enduring viral nucleic acid suppression; however, a high toxicity is a burden and negative factor for clinical application [19,20]. GLS4, which shows similar effective concentrations, is only slightly less toxic [21]. Therefore, it is important to find nucleocapsid modulators that are efficient and less toxic. Currently, several new HAP compounds, e.g., R10 and RG7907(R07049389), are promising candidates for clinical trials [22,23].

The molecular mechanism of action of CAMs is still unclear. To avoid the confusion in the literature about class I versus class II CAMs, depending on the mechanism of action [24,25], we will further use the nomenclature proposed by Wang S et al. [26]. The CAM-N class refers to modulators that cause normal capsid structures, and the CAM-A class refers to modulators that cause abnormal capsid structures. HAPs belong to the CAM-A group, binding to a shallow, understated hydrophobic pocket (unlike enzymes and receptor pockets, which are usually much deeper) at the dimer–dimer protein interface. Another group of HBV inhibitors that do not induce aberrant capsid structure formation but influence nucleic acid packaging/release, CAM-N compounds (e.g., sulfamoylbenzamides), also bind the same pocket. On the other hand, it was discovered that HAP binding could be different and these compounds can also bind to a hydrophobic sub-pocket [27].

Structural studies with nucleocapsid assembly modulators have mostly been performed using a shortened HBc protein with a cleaved C-terminal domain expressed in *E. coli*. HBc self-assembles into capsids, and it is possible to ensure their assembly and disassembly under controlled in vitro conditions by adding capsid assembly-inducing compounds in known molar ratios and performing capsid assembly dynamics and structural studies [28]. However, the self-assembly process of the shortened HBc protein and the build-up of capsids could be different from the full-length HBc proteins found in the natural environment. Recently, after resolving the structure of several previously unresolved residues of the HBc C-terminal domain that precedes the phosphorylation sites, it was found that these residues can increase the inter-dimer contacts. Thus, the C-termini likely play an important role in capsid stabilization and provide a much larger interaction interface than was previously observed [29].

We previously developed and optimized the use of the recombinant alphavirus expression vector for effective target protein synthesis within 24 h [30,31], which cannot be achieved in standard HBV-producing mammalian cell cultures [32]. Here, we applied the alphavirus-based expression of full-length HBc gene and performed the assessment of HBV capsid assembly in a natural mammalian cell environment, allowing the direct selection of capsid modulators. A set of compounds were designed and synthesized by introducing modifications to several groups of the HBV CAM-A reference compound Bay 41-4109. All studied compounds were used as racemic (rac) mixes. The most efficient compounds were the dihydropyrimidine-5-carboxylic acid alkyl esters, some of which showed low toxicity and induced concentration-dependent inhibition of capsid formation. Depending on compound chemical structure elements (features) accumulation of aberrant capsid assembly products were revealed by changes in native agarose gel electrophoresis mobility and confirmed by electron microscopy.

## 2. Results

### 2.1. Chemistry

**Figure 2:**
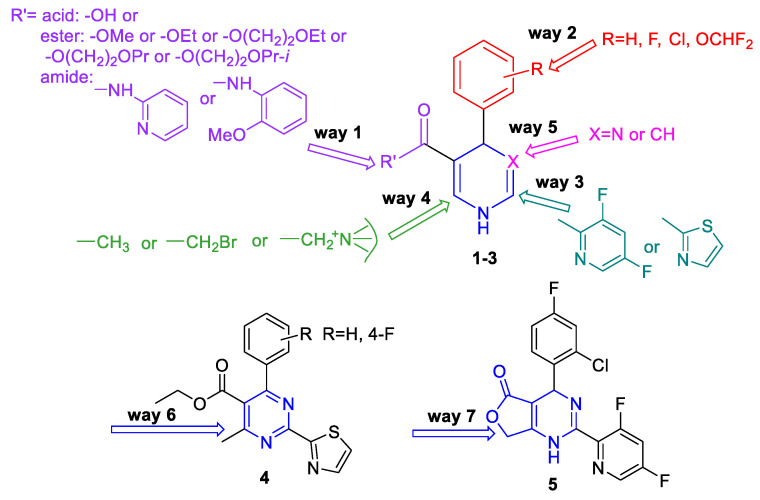
Structural diversifications of heteroaryldihydropyrimidines.

**Figure 3:**
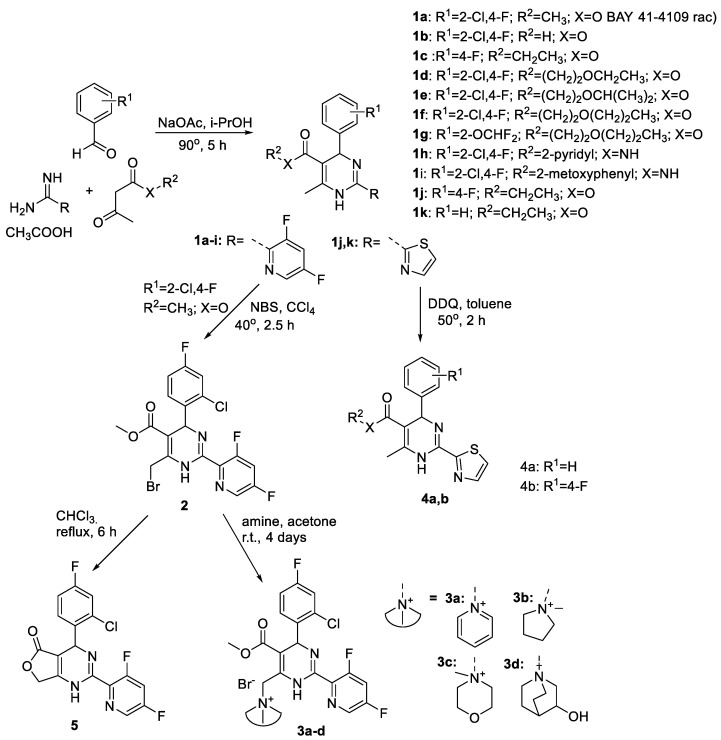
Synthesis of heteroaryldihydropyrimidine derivatives.

**Figure 4:**
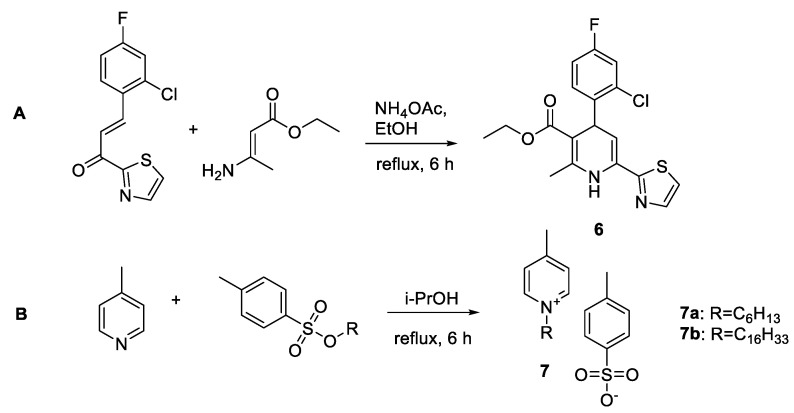
(**A**) Synthesis of heteroaryldihydropyridine **6** and (**B**) 1-alkyl-4-methylpyridin-1-ium 4-methylbenzenesulfonates **7**.

HAPs were shown previously to bind HBV core protein [33]. Taking into consideration the structure–activity relationship data, structural diversifications based on HAP compound Bay 41-4109 (compound 1a, see also Table 1A) and its analogues were carried out in this study (Figure 2). Synthetic pathways of the groups of HAP derivatives and closely or less closely related analogues were elaborated (Figure 3 and Figure 4). The details of the synthesis and physicochemical characterization of new compounds are provided in Supplementary material. Representative novel compounds **1b-j**; **3a-d**; **4a,b**; **5**; **6** were synthesized in order to study the misdirection of HBV capsid self-assembly, see Table 1A,B for summarized details.

The design of new molecules was performed using targeted derivatization of parent compound Bay 41-4109 (comp. **1a**) by adding or subtracting functional groups in following ways (Figure 2):

(1) Changes at position 5 of the dihydropyrimidine core led to compounds containing carboxylic acid moiety (comp. **1b**), ester moiety (comp. **1c-1g** and **1j-1k,** including alkoxyalkyl esters **1d-1g**), and amide moiety (including anilide **1h** and pyridylamide **1i**).
(2) Variations at position 4 of the dihydropyrimidine core led to the formation of compounds with phenyl and substituted phenyl (including 4-fluoro, 2-difluoromethoxy, and 2-chloro-4-fluoro substituents) groups.
(3) Derivatization at position 2 of the dihydropyrimidine core led to 3,5-difluoropyridyl-2 (comp. **1a-1i**) or thiazolyl-2 (comp. **1j-1k**) group containing analogues.
(4) Insertion of bromine atoms or cation-substituted ammonium groups at position 6 of the dihydropyrimidine core led to compounds **3a-3d**.
(5) Dihydropyridine **6** (or deaza-HAP) was synthesized via the cyclocondensation reaction of the aminovinylcarbonyl and arylidene carbonyl compounds.
(6) Heteroarylpyrimidines **4a** and **4b**—pyrimidine derivatives as oxidized HAP representatives were obtained by aromatization of comp. **1j** and **1k**.
(7) Additionally, derivative at positions 5 + 6 (condensed furanone cyclic comp. **5**) was obtained by heating of 2-bromomethyl-HAPs compound.

The synthesis of HAPs **1a-k** was performed under Biginelli conditions. Reaction of an appropriate amidine, aromatic aldehyde, β-ketoester or N-substituted carboxamide and NaOAc in izopropanol gave compounds **1a-k** as racemic mixtures (Figure 3).

To synthesize cationic moiety containing comp. **3a-d** and bicyclic dihydropyrimidine **5**, bromination of parent dihydropyrimidine **1a** was performed with N-bromosuccinimide (NBS) in CCl_4_ solution. After which, the obtained bromide derivative **2** was further treated with tertiary amine giving **3a-d**. Lactone **5** was obtained by refluxing of of bromide **2** in CCl_4_. Cationic moiety containing HAPs have not been studied and published so far as HBc capsid modulators. On the other hand, the nitrogen containing heterocycles in position 6 of dihydropyrimidine core are known to increase the activity of compounds [20,22]. HAPs derivatives **1j,k** were oxidized with DDQ in toluene to appropriate pyrimidines **4a**, **4b** using a previously published method for oxidation of GLS4 [34].

The deazaheteroaryldihydropyrimidine analogue (heteroaryldihydropyridine) **6** was synthesized according to the scheme in Figure 4A by the cyclocondensation of ethyl (E,Z)-3-aminobut-2-enoate with 4-(2-chloro-4-fluorophenyl)-1-(thiazol-2-yl)propanone, which was obtained in situ from 2-chloro-4-fluorobenzaldehyde and 2-acetylthiazole.

As new analogues of cetylpyridinium chloride, a recently announced HBV capsid assembly modifier [35] N-alkylpicolinium tosylates **7a** and **7b** were synthesized from4-picoline and appropriate hexyl- or hexadecyl 4-methylbenzenesulfonate in 2-propanol according to scheme depicted in Figure 4B.

The purities of the studied compounds were at least 98% according to high-performance liquid chromatography data.

### 2.2. Evaluation of the Compound Effects on HBV Capsid Assembly in HBc-Producing BHK-21 Cells

To define HAP compound non-toxic concentrations for initial studies, non-infected BHK-21 cells were incubated with compounds at four different concentrations for 24 h (25 μM, 50 μM, 100 μM, and 200 μM), and the cytopathic effects were evaluated by analysis of cell morphology and proliferation changes. Different toxicity profiles were observed for tested compounds, with some compounds being less toxic than others. The highest compound concentrations that did not induce noticeable cytopathic effects in treated BHK-21 cells are listed in Table 1A,B (the highest non-toxic concentration, μM). Low toxicity (200 μM and higher) exhibited the comp. **1d**, **3a**, **3b**, **4b**, and **5**, whereas high toxicity (25 μM and lower) was observed for comp. **1c**, **1j**, **1k** and **4a**, and **7b**. The reference comp. **1a** (Bay 41-4109) revealed cell toxicity signs at 100 μM concentration.

**Figure 5:**
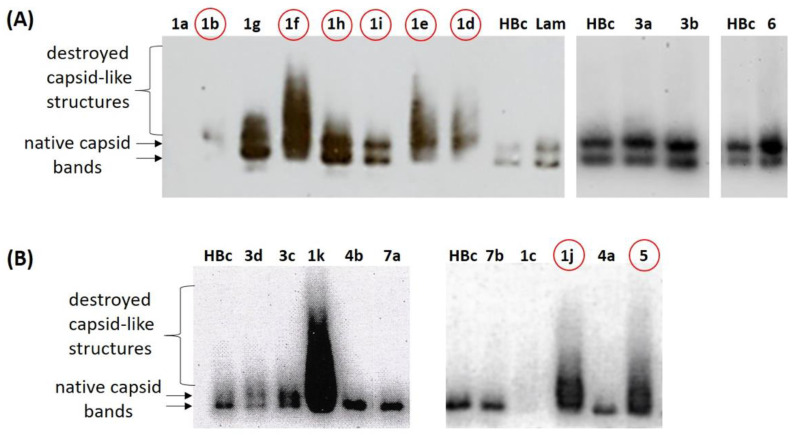
Analysis of the effects of HAP compounds on assembly of HBV capsids in mammalian cells. BHK-21 cells were infected with SFV1/HBc (D1 genotype) to express full-length HBc gene. After 4 h, the cell culture medium was replaced with compound-containing medium, and after 24 h of cell incubation with respective compound the medium was removed and the cell lysates were analyzed by native agarose gel electrophoresis and subsequent anti-HBc immunoblot. (**A**) Cells treated with compounds: **1a** (Bay 41-4109), 10 µM; **1b**, 10 µM; **1g**, 10 µM; **1f**, 10 µM; **1h**, 10 µM; **1i**, 10 µM; **1e**, 10 µM; **1d**, 10 µM; **HBc**, non-treated HBc producing BHK-21 control cells; **Lam** (lamivudine), 10 µM; **3a**, 20 µM; **3b**, 20 µM; **6**, 10 µM. (**B**) Cells treated with compounds: **HBc**, non-treated HBc producing BHK-21 control cells; **3d**, 50 µM; **3c**, 50 µM; **1k**, 10 µM; **4b**, 50 µM; **7a**, 25 µM; **7b**, 1 µM; **1c**, 5 µM; **1j**, 5 µM; **4a**, 5 µM; **5**, 50 µM. Capsid bands are indicated with arrows; capsid-like structures are indicated with brace. Equal protein amount of the total cell lysates was loaded in each well of the agarose gel. Compounds selected for further dose-dependent study are indicated in red circles.

After the initial toxicity evaluation, the compounds were tested at non-toxic concentrations in infected HBc-producing BHK-21 cells for their ability to influence capsid production. For this purpose, BHK-21 cells were infected with pSFV1/HBc vector expressing full-length HBc gene of the genotype D1, as described in Methods, Section 4.6. These HBc-producing BHK-21 cells were treated with studied compounds in a range 1–50 μM concentration for 24 h. The assembly of the capsids was evaluated by native agarose immunoblots, allowing detection of assembled core particles and their intermediate forms under non-denatured conditions (Figure 5). The untreated HBc-producing BHK-21 cells were used as a control, and the Bay 41-4109 (comp. **1a**) was used as a reference inhibitor. All synthesized compounds were tested. As a result, several compounds led to misfolding of the HBV capsid, producing a smear of the high molecular weight HBc structures in agarose gel. These comp. **1d**, **1e**, **1f**, **1j**, **1k**, and fused comp. **5**, potentially may affect the formation of native HBV capsids and prevent their envelopment. Comp. **1b**, **1c**, and **3d** showed inhibitory effect. Interestingly, many of the tested compounds led to the accumulation of core particles (capsids), which is evident from the increased intensity of the HBc signal, allowing to assume a mechanism of action different from Bay 41-4109 (comp. **1a**). Eight compounds that induced changes in capsid signal distribution (Figure 5, indicated in red circles) were further tested in dose-dependent experiments: **1b**, **1d**, **1e**, **1f**, **1h**, **1i**, **1j**, and **5**. Comp. **1c**, **1j**, and **1k** were excluded due to the observed considerable toxic effects on the cells. Comp. **1g**, **3a**, **3b**, **3c**, **4a**, **4b**, **6**, **7a**, and **7b** did not induce a significant effect on the capsid mobility.

**Figure 6:**
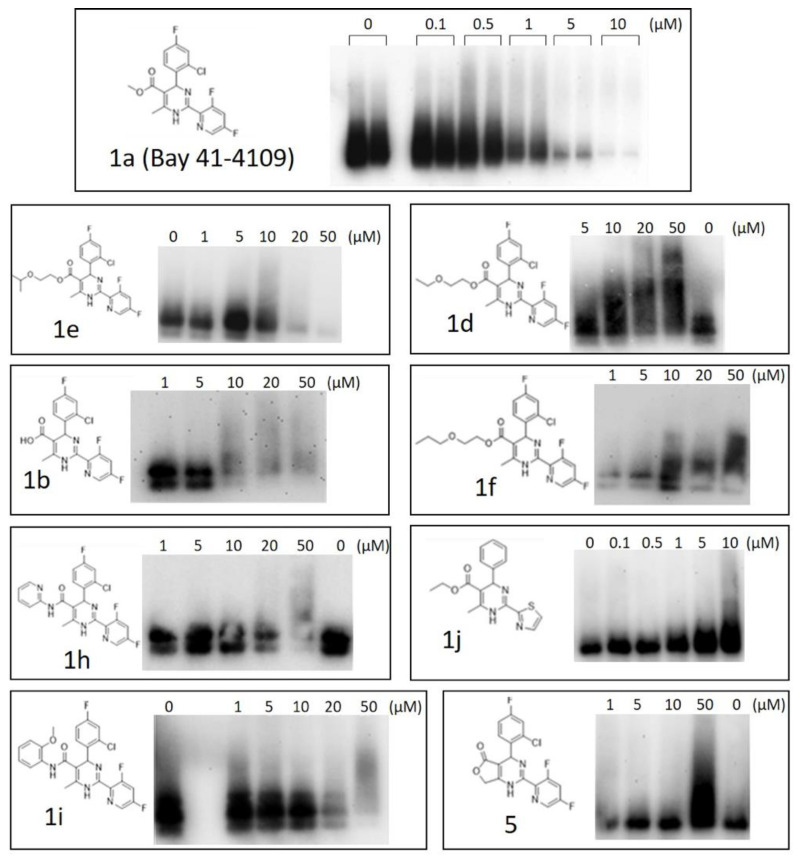
Dose-dependent effects of selected compounds on the assembly of HBV capsids in mammalian cells. Native agarose gel electrophoresis and subsequent anti-HBc immunoblotting of the cell lysates is shown. BHK-21 cells were infected with SFV1/HBc (D1 genotype) to express full length HBc gene. After 4 h, the cell culture medium was replaced with compound-containing medium, and after 24 h of incubation the cell medium was removed and the lysates were loaded on agarose gel. Equal total protein amount was tested. The concentrations (in a range 0.1–50 µM) are indicated on the top of each immunoblot. The concentration “0” (without compound) for comp. **1b** test corresponds to the line “0” in comp. **1h** immunoblot; the concentration “0” for comp. **1f** test corresponds to the line “0” in comp. **1d** immunoblot, because it was the same run for both compounds with shared untreated HBc control.

Practically, two type of effects on HBV capsid assembly were observed in dose-dependent experiments (Figure 6): (i) a dose-dependent HBc signal decrease, presumably due to degradation by cells, as was detected for comp. **1a** (Bay 41-4109), **1b**, **1e**, **1h**, and **1i** (Figure 6, on the left). (ii) a dose-dependent HBc products accumulation, as was detected for comp. **1d**, **1f**, **1j**, and **5** (Figure 6, on the right).

In summary, from the results obtained in native agarose immunoblot experiments, the unexpected HBc accumulation was observed as follows:

(i) By prolongation of the methyl ester group at position 5 of the Bay 41-4109: 5-ethoxyethoxycarbonyl **1d** and 5-propoxyethoxycarbonyl **1f**, that is, in the case of n-alkoxyalkylcarbonyl substituents. In the case of small deviations in the structures of comp. **1d** and **1f**, comp. **1e**, containing an i-propoxyethoxycarbonyl moiety at position 5, did not lead to HBc accumulation; in contrast, a dose-dependent capsid signal decrease was observed. Comp. **1g**, a close analogue of comp. **1f**, comprising an o-difluoromethoxyphenyl group in position 4 of the dihydropyrimidine molecule, showed some increase in capsid band signal (Figure 5);
(ii) In the case of lactone derivative **5** of the acid **1b**;
(iii) In the case of 2-(thiazolyl-2)-5-ethoxycarbonyl-6-methyl-1,4-dihydropyrimidones containing 4-phenyl and 4-(4-fluoro)-phenyl substituents (comp. **1j** and **1k**).

### 2.3. Cytotoxicity Estimation of Selected Compounds in BHK-21 Cells with the MTT Viability Assay

For selected 2-(3,5-difluoropyridyl-2)-4-(2-chloro-4-fluorophenyl-4)-1,4-dihydropyrimidine derivatives (comp. **1b**, **1d**, **1e**, **1f**, **1h**, and **1i**) that showed remarkable effect on the self-assembly of the HBc protein in the BHK-21 model and low toxicity by the cell morphology examination and trypan blue stain exclusion, further MTT-based cell viability testing was performed. The comp. **1j** and **5** were not selected due to the absence of significant dose dependent inhibitory effects, which instead showed the accumulation of the HBc capsid-like structures.

**Figure 7:**
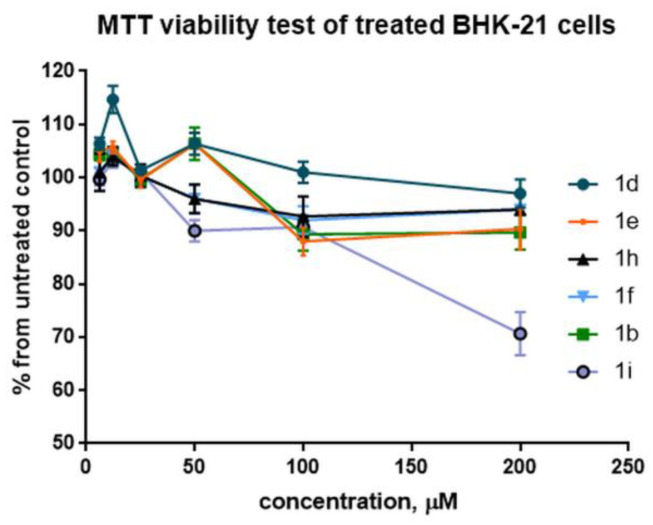
MTT viability test of BHK-21 cells treated with selected HAP compounds **1b**, **1d**, **1e**, **1h**, **1f**, and **1i**. The BHK-21 cells were incubated with medium containing the respective amount of the compound (6.125; 12.5; 25; 50; 100; 200 μM) for 24 h, then the cell viability was measured by MTT-based assay. The results are shown as a percentage of optical density (OD) units compared with the non-treated control BHK-21 cells (100%); error bars represent the standard deviation of three repeats.

BHK-21 cells were incubated with different concentrations (6.125, 12.25, 25, 50, 100, and 200 µM) of the respective compounds and the cell viability was calculated as a percentage from untreated cells (100%) (Figure 7). In general, the results confirmed the morphological observation of relatively low cytotoxicity for tested compounds at concentrations below 100 µM. The lowest cytotoxicity was observed for comp. **1d**, which did not demonstrate the difference in cell viability comparing to untreated control cells. The highest toxicity was detected for compound **1i** at 200 µM concentration (71% viable cells comparing to the untreated control), whereas the viability of cells treated with compounds **1b**, **1e**, **1f**, and **1h** did not change significantly (>90% at 200 µM).

### 2.4. Antiviral Effects Study in HBV-Producing HepG2.2.15 Cells

Four HAP compounds—**1b**, **1d**, **1e**, and **1f**, which showed both an inhibitory effect of the HBc protein self-assembly and a low cytotoxicity, were selected for analysis of their antiviral effects in HBV producing hepatocellular carcinoma cell line. HepG2.2.15 cells [36], stably producing HBV virions, were incubated with respective compounds for six days, as described in methods, and the effect of each compound on HBV virion secretion was determined by isolating the HBV DNA from secreted virions in cell media followed by real-time PCR DNA quantification. As HBV production in HepG2.2.15 cell culture may vary depending on the growth conditions [32], the culture conditions were verified and optimized prior to antiviral effect testing. Internal DNA controls were used for optimal viral DNA isolation and quantification (see Methods, Section 4.7 and Section 4.8). DNA isolated from untreated HepG2.2.15 cell medium was used as a control for quantification.

**Figure 8:**
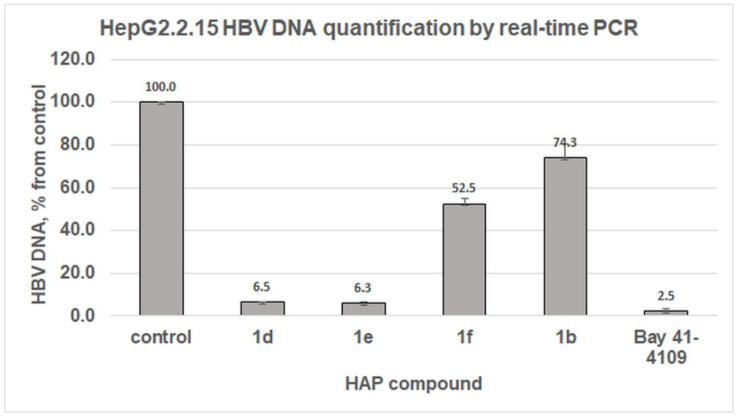
Quantification of HBV DNA in HepG2.2.15 HBV-producing cells treated with HAP compounds. HepG2.2.15 cells were cultured in the presence of the studied HAP compounds in 24-well plates for six days: comp. **1d**, **1e**, **1f** (20 μM), and comp. **1b**, Bay 41-4109 (10 μM). After incubation the cell medium was collected, the virus protected HBV DNA was isolated and quantified by real-time PCR analysis. Untreated cells were used as a control (100%); error bars represent the standard deviation of three repeats.

As a result of the quantitative determination of HBV DNA, comp. **1d** and **1e** at concentration 20 µM strongly inhibited HBV virion secretion and reached only 6.5% and 6.3%, respectively, compared with the untreated HepG2.2.15 cells (100%), whereas comp. **1f** (20 µM) and **1b** (10 µM) had moderate inhibitory effects, 52.5% and 74.3%, respectively (Figure 8). The reference compound Bay 41-4109 (comp. **1a**) (10 µM) significantly inhibited HBV virion secretion and led to 2.5% of virion secretion comparing to the untreated control. 

**Figure 9:**
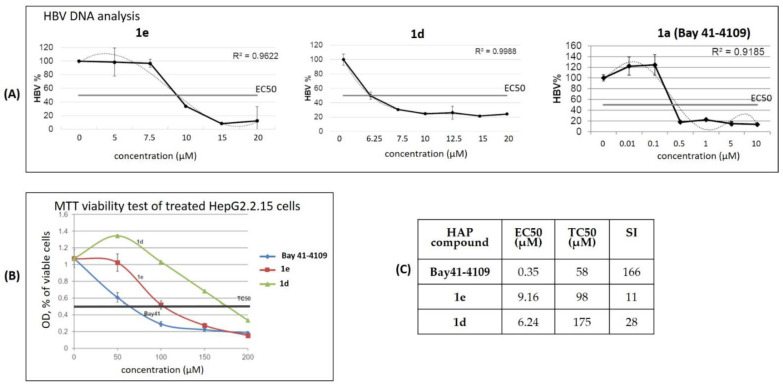
Antiviral activity and cell viability analysis of HAP comp. **1e** and **1d** in comparison with the reference comp. **1a** (Bay 41-4109). (**A**) The HepG2.2.15 cells were cultured with the respective concentration of tested compound for six days. The HBV DNA was isolated from secreted virions from cell medium and quantified by the real-time PCR. Untreated cells were used as a control (100%); error bars represent the standard deviation of three repeats. (**B**) MTT-based viability test of HepG2.2.15 cells treated with respective compound for 48 h. Untreated cells were used as a control (100%); error bars represent the standard deviation of three repeats. (**C**) Calculated TC_50_ and EC_50_ concentrations and the corresponding selectivity indices SI (TC_50_/EC_50_ relationship) of comp. **1a** (Bay 41-4109) **1e**, **1d**.

To characterize the antiviral capacity of the discovered active compounds **1d** and **1e**, the EC_50_ values (median effective concentration) were calculated. For this purpose, HepG2.2.15 cells were treated with six different concentrations of HAP compounds (in a range 0–20 µM) for six days and the real-time PCR analysis of the amount of HBV DNA extracted from virions was performed. Using the data obtained from each sample, trend curves were created, and the effective concentration of the inhibitor required to reduce the HBV DNA by 50% was calculated for comp. **1d**, **1e** and Bay 41-4109 (Figure 9A,C). Although the most efficient EC_50_ concentration was detected for the reference comp. **1a** (0.35 µM), the tested comp. **1d** also has demonstrated a remarkable inhibitory concentration (6.24 µM) that together with the low cytotoxicity could represent promising properties for HBV assembly inhibition.

In order to compare the cytotoxicity of compounds **1d** and **1e** in HBV producing HepG2.2.15 cells, the toxic concentrations (TC_50_ values) were estimated (Figure 9B,C). The MTT viability test was performed in HepG2.2.15 cells treated with different concentrations (0–200 µM) for 48 h, and the mean TC_50_ values were calculated for each compound. As was expected, comp. **1d** showed the highest viability dose (175 µM), whereas the control comp. **1a** (Bay 41-4109) was more than three times toxic (58 µM). Nevertheless, the corresponding selectivity index (SI), defining the TC_50_/EC_50_ value, for the Bay 41-4109 was higher (SI = 166), than for the tested compounds **1d**, **1e** (SI = 28 and SI = 11), respectively.

### 2.5. Evaluation of the HBc Polymer Structures Induced by Compound **1d** in BHK-21 Cells

It was clear from the native agarose immunoblot experiments that the comp. **1d** provoked noncanonical assembly of HBc protein into polymer structures and induced their accumulation in cells. Previous studies showed that Bay 41-4109 induces the formation of aberrant structures in vitro after incubation with purified HBc dimers produced in *E. coli* [10]. However, in mammalian cells, HBc polymer structures were not detected for Bay 41-4109 in native agarose electrophoresis, as was shown by other authors [37,38] and confirmed in this study as well (Figure 5 and Figure 6). Presumably, the incorrect HBc polymer structures are efficiently degraded by the cells, which was not a case for comp. **1d** inducing the accumulation of HBc. The accumulation of HBc within the cell and the intracellular transport plays a significant role in HBV replication and pathogenesis [39,40]. Using confocal microscopy, we have investigated the intracellular localization of HBc in cells treated with comp. **1d**.

**Figure 10:**
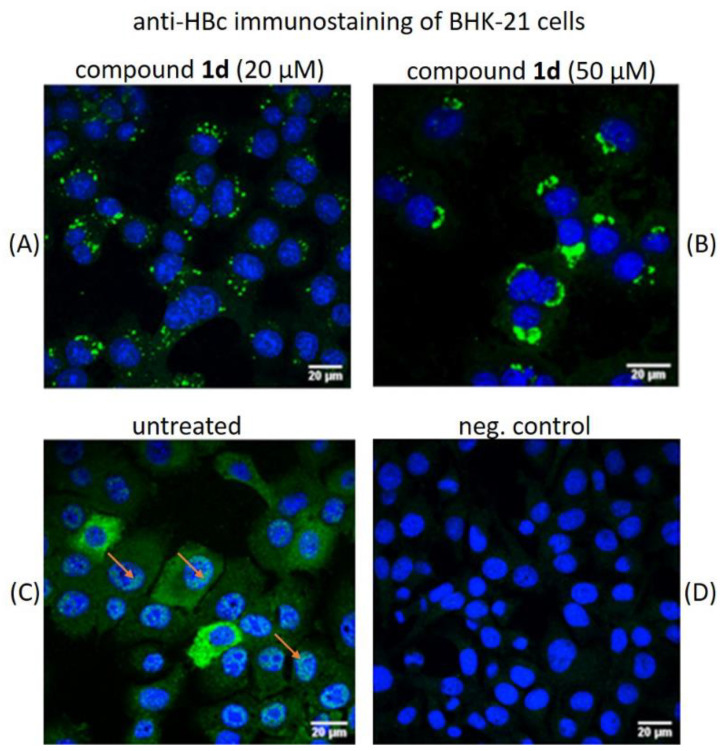
Immunostaining of HBc protein in BHK-21 cells after treatment with HAP compound **1d**. BHK cells were infected with recombinant SFV1/HBc virus, next day the cell culture medium was replaced with comp. **1d** containing medium and incubated for 24h. After incubation the anti-HBc immunostaining analysis was performed using monoclonal C1-5 antibodies (green fluorescence). The cells were analyzed by confocal microscopy and compared with untreated cells. The nuclei were stained with DAPI (blue). (**A**) cells treated with 20 µM comp. **1d**. (**B**) cells treated with 50 µM comp. **1d**. (**C**) Untreated cells; the orange arrows indicate the nuclear localization of HBc in untreated cells. (**D**) Negative control represents BHK-21 cells uninfected with SFV1/HBc virus and untreated with the compound.

HBc-producing BHK-21 cells were treated with 20 μM and 50 μM of comp. **1d** for 24 h, and the confocal immunocytochemistry microscopy analysis was performed. Cell staining with monoclonal anti-HBc antibodies showed a speckled dot pattern of HBc distribution in the cytoplasm of comp. **1d** treated cells, which was clearly different from the more homogenous allocation of HBc in both the cytoplasm and the nucleus of the untreated HBc-expressing BHK-21 cells (Figure 10). This specific pattern was also observed by performing staining with rabbit polyclonal anti-HBc antibodies (Dako, cat. No. B058601), data not shown. Remarkably, the cell treatment with comp. **1d** blocked the intranuclear transport of HBc protein, which, on the contrary, was found in untreated cells (Figure 10C, arrows). Furthermore, the higher concentration of comp. **1d** induced higher accumulation of HBc protein products in cell cytoplasm (Figure 10B).

In order to investigate the morphological properties of HBc assembly structures, the electron microscopy analysis was performed. The capsid-like structures were purified from the lysate of HBc-expressing BHK-21 cells after treatment with comp. **1d**. For this purpose, the capsid-like structures from the lysate were pelleted by ultracentrifugation through a 20% sucrose cushion, then resuspended in buffer and subjected for electron microscopy. The cell lysate from untreated cells was processed in similar way and used as a control of HBc particles.

**Figure 11:**
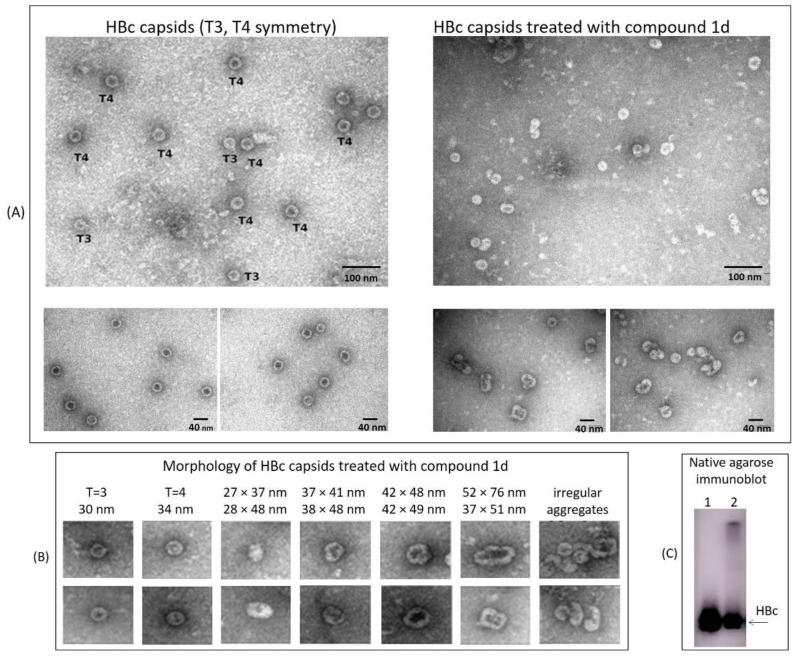
Electron microscopy analysis of particles concentrated from the lysates of BHK-21 cells after treatment with comp. **1d**. BHK-21 cells were infected with pSFV1/HBc to allow HBc protein expression. Three hours post-infection, 50 μM comp. **1d** was added to cell medium, and the cells were further incubated for 24 h. After incubation the cell lysates were performed and used for ultracentrifugation through 20% sucrose cushion. The pellets containing capsids and high molecular capsid-like structures were resuspended and used for electron microscopy analysis (TEM). (**A**) Electron microscopy pictures of regular HBV core particles of standard T3 and T4 symmetry isolated from untreated cells (on the left) and irregular misassembled core particles isolated from cells treated with comp. **1d** (on the right). (**B**) Selected HBc protein assembly products visible by electron microscopy from cells treated with HAP comp. **1d**. The approximate particle size was measured as the longer and shorter diameter of the irregular form of the particle. (**C**) Native agarose anti-HBc immunoblot of samples prepared for electron microscopy: line 1—untreated HBc; line 2—HBc treated with comp. **1d**.

The electron microscopy of comp. **1d** treated samples revealed the presence of structures that were different from the standard T = 4 or T = 3 symmetry capsids found in the control preparation, which varied in size and shape (Figure 11A). Electron microscopy observations of the capsids from transduced BHK-21 cells without treatment corresponded in size and shape to standard T = 3 and T = 4 symmetric capsids. HBc assembly products from cells treated with comp. **1d** showed rare T = 3 and T = 4 capsids of the appropriate size, 30 nm and 34 nm, respectively; notably, irregularly shaped structures that resembled capsids of the wrong size and incorrect symmetry, as well as the capsid aggregates, were detected in the comp. **1d** treated preparation (Figure 11B). These capsid-like products have different diameters, irregular morphology and larger opened compositions. Interestingly, both purified through sucrose cushion samples (untreated and treated with comp. **1d**) revealed similar bands with close electrophoretic mobility in native agarose immunoblot, except some accumulation of HBc specific signal at start position for the comp. **1d** treated capsids (Figure 11C).

### 2.6. Molecular Modeling of HAP Compound Interaction with HBc Protein

In order to understand the mode of interaction and rationalize the structure–activity relationships (SAR) of the studied compounds, molecular docking was performed for selected compounds: standard comp. **1a**; four compounds accumulating HBc and leading to incorrect capsid structures (two compounds possessing 3,5-difluoropyridyl substituent in position 2 of the dihydropyrimidine cycle—**1d** and **1f**, and two compounds having thiazolyl ring in the same position—**1j** and **1k**); comp. **1e**—possessing close structure to **1d** and **1f**, but decreasing capsid signal; three compounds lacking effect on the assembly of HBV capsids (**3b** having cationic ammonium methyl moiety in position 6 of the HAP, two derivatives of heteroaromatic pyrimidine **4a** and **4b**).

The compounds were docked using the Schrödinger software package release 2018-4 induced fit docking (IFD) simulation protocol. (i) Two pairs of active/inactive compounds related to accumulation of capsid proteins in the cells were selected (comp. **1j** versus **4b** and **1k** versus **4a**), each pair has the same carbon—nitrogen—oxygen skeleton, the only difference is the dihydropyrimidine system (comp. **1j** and **1k**) or relative heteroaromatic pyrimidine system (comp. **4b** and **4a**). (ii) Comp. **1a** comprising activity to decrease capsid signal and analog comp. **3b** having positive charged pyrrolidinium substituent in position 6 were analyzed. (iii) Three comp. **1d**, **1e**, and **1f** having alkoxyethylester substituents in position 5 of dihydropyrimidine system were analyzed. Comp. **1d** and **1f** have ethoxyethyl ester and *n*-propoxyethyl ester substituents in position 5, but comp. **1e** has *i*-propoxyethoxy ester substituent in position 5. Comp. **1d** and **1f** promote HBc accumulation or formation of aggregates or incorrect capsid structures. Quite close comp. **1e** leads to dose dependent capsid signal decrease.

**Figure 12:**
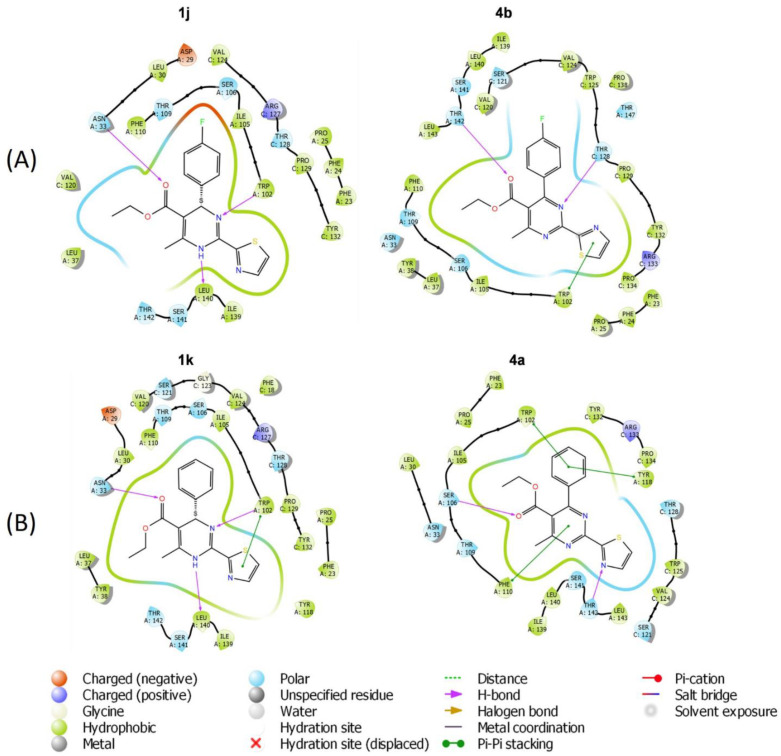
Interaction diagram of HAP compounds docked to HBc dimer interface. (**A**) Compounds **1j** and **4b**. (**B**) Compounds **1k** and **4a**.

The docking results showed that hydrogen bonds with residues Trp102 and Leu140 located in the HBc dimer interface are important for inhibitor binding. The binding pocket adjusts shape by induced fit upon the inhibitor binding; thus, it was feasible to dock large compounds with bulky substituents. The docking scores did not show a quantitative correlation with the experimental data, but it was possible to investigate the differences in the binding poses of the compounds. Comparison of the binding poses of comp. **1j** and **4b** (Figure 12A) showed that the compound containing a pyrimidine ring (**4b**) cannot form hydrogen bonds with Trp102 because the compound does not bind in the same conformation as 1,4-dihydropyrimidine series of compounds. The same conclusion was drawn after comparison of the binding of comp. **1k** and **4a** (Figure 12B). In agreement with these results, dihydropyrimidines **1j** and **1k** induced the accumulation of capsid proteins in the cells, but pyrimidines **4a** and **4b** lacked significant anti-HBc activity.

**Figure 13:**
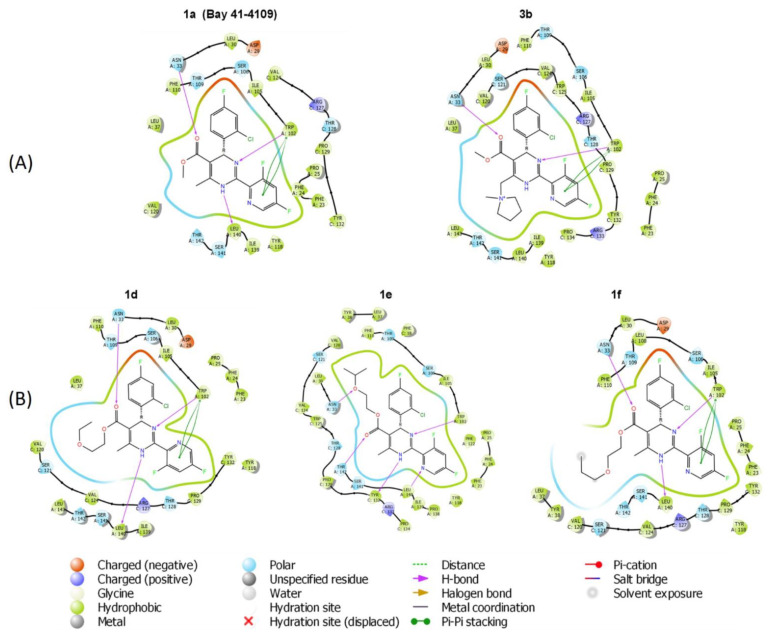
Interaction diagram of HAP compounds docked to HBc dimer interface. (**A**) Compounds **1a** and **3b**. (**B**) Compounds **1d**, **1e** and **1f**.

The addition of a large substituent at position 6 of the 1,4-dihydropyrimidine ring disturbs the formation of H-bonds between the NH at position 1 and Leu 140 (Figure 13A). As a result, comp. **3b** lacks activity comparing to comp. **1a** (Bay 41-4109). Moreover, the positive charge of the substituent at position 6 can be an additional disturbing factor. Interestingly, comp. **1d**, **1e**, and **1f** with alkoxyalkyl ester substituents at position 5 of the 1,4-dihydropyrimidine ring showed almost similar docking poses (Figure 13B) with the small differences for the comp. **1e** comparing to comp. **1d** and **1f**: **1e** is not making Pi–Pi stacking with Trp10; Leu140 is forming H-bond with pyridine nitrogen and not with NH at position 1 of the 1,4-dihydropyrimidine; Asn33 is not forming the H-bond with the carboxyl group. However, the current IFD model could not comprehensively describe the differences in activity (comp. **1e** did not induce a remarkable capsid accumulation in the cells compared with comp. **1d** and **1f**) and could show only the binding at the HBc dimer interface. Thus, there are other influencing factors that are not included in the IFD model.

## 3. Discussion

Chemotherapy of viral infections has to selectively reduce virus-specific processes that proceed in a cell without any influence on cell metabolism itself. This essentially explains the limited set of antiviral drugs applied in clinical practice. Viral capsids are unique virus-specific structures; therefore, capsid modifiers do not have other adequate targets in eukaryotic cells and subsequently, fewer side effects are expected in the clinic.

Partially hydrogenated pyrimidines (Bay 41-4109, GLS4) have been shown to target the capsid protein of HBV, diminishing the stability of the capsids and enhancing the rate of HBc association [41]. Depending on the structure of the agent, the route of exposure or the binding site, multiple effects on HBV capsid assembly dynamics and stability may occur.

CAMs-A, such as Bay 41-4109 and GLS4, misdirect capsid assembly to form non-capsid core protein polymers [17,41,42]. All of the other reported CAMs, including sulfamoylbenzamides, benzamides, and phenylpropenamides, induce the formation of morphologically normal capsids of variable sizes that are devoid of viral pgRNA and DNA polymerase and are thus categorized as CAMs-N [43,44].

Despite strong antiviral effects, Bay 41-4109 and GLS4 have demonstrated significant hepatotoxicity problems. In rat model, the NMR spectroscopy analysis of urine, serum, and liver tissue extracts revealed triglyceride terminal methyl, methylene groups and CH_2_CO, N^+^(CH_3_)_3_, CH_2_OPO_2_, and CH_2_OCOR accumulation [45]. These observations provided evidence that fatty acid metabolism disorder and mitochondrial inability might contribute to the hepatotoxicity of Bay 41-4109. 1,4-Dihydropyrimidine derivatives and Biginelli type compounds may reveal antioxidant activity [46,47].

Morphological and trypan blue staining examinations of the cytopathic effects in BHK-21 cells treated with our studied compounds showed that the addition of lipophilic radicals at position 5 substantially diminished the toxic cellular effects, as observed in the case of comp. **1d**, **1e**, and **1f** versus **1b** (Table 1). After cell treatment with comp. **1d**, cells at 80% confluency showed no toxic effects up to 200 μM concentration. Comp. **1d** was clearly less toxic than Bay 41-4109 in HepG2.2.15 cells after 48 h of incubation, as shown by the MTT assay and calculated TC_50_ values (Figure 8B,C). The effects of the comp. **1d** on cellular metabolism at the higher concentration (>200 μM) was difficult to assess because the compound was dissolved in DMSO, which can be toxic to many cells at high concentrations [48]. The toxic concentration of Bay 41-4109 varies according to different authors; for example, in primary hepatocytes, Bay 41-4109 was toxic at a concentration of 5.06 μM [49]. Moreover, the testing of the long-term incubation is necessary to assess potential accumulated cell damage.

The mechanism of abnormal HBV core protein assembly triggered by HAPs is still not fully understood, and the detailed interactions between capsids and different classes of inhibitors remain elusive, hampering the rational design of drugs for assembly dysregulation. To understand the defining principles of the obtained experimental data, we performed molecular docking analysis using the full-length HBc molecule as a homology template. The results clearly revealed for comp. **3b**, **4a**, and **4b** that the ability of the aromatic ring to form hydrogen bonds with the amino acids tryptophan or leucine at the HBc hydrophobic dimer interface is crucial for compound anti-HBc activity, and compounds with oxidized dihydropyrimidine moieties lack this specific activity in accordance with the experimental data. Current molecular docking model does not allow the detailed prediction of the effects induced by HAP-derived compounds. Considering comp. **1d**, **1e**, and **1f**, which contain alkoxyethoxycarbonyl substituent at position 5 of the 1,4-dihydropyrimidine ring, similar SAR data were observed including similar docking poses and anti-HBc modulating activity (Figure 6 and Figure 13). Although the IFD model for these promising compounds was also similar to Bay 41-4109 IFD model in terms of hydrogen bond formation (Asn33, Trp102, and Leu140) and overall docking pose, current SAR approach cannot explain the biological differences between these compounds: HBc accumulation, aggregation, formation of irregular structures. In this case, to explore SAR trends, more structural data and a detailed biological profiling are required.

The lack of adequate HBV infection models still hinders the discovery of new therapeutic strategies to eradicate HBV and cure chronic infection. The activity of HBV antivirals is usually tested using stably transfected hepatoma-derived cell lines with integrated genotype D, such as HepG2.2.15 and HepG2.117 or HB611 cells [50]. The HepG2.2.15 cell line has been developed as a standardized method, representing a model for chronic cellular HBV replication studies and in vitro screening for antiviral compounds. These model cell lines produce infectious HBV virions, which require high-grade safety standards. Additionally, the synthesis of HBV is relatively inefficient, and cells have to be cultivated approximately 5–7 days before assessment of the antiviral effects of a compound. Moreover, various authors have stated that anti-HBV activity differs according to the genetic variant of the capsid-forming HBc protein gene. Different clinical isolates show different results for Bay 41-4109 in efficacy trials, there are also differences between HBV genotypes observed [51,52]. The availability of HBV stably transfected cell lines for investigation of different genotypes and their variants is limited. Therefore, a new approach for efficient modeling of HBV assembly is required.

Previously, we developed and optimized the use of the recombinant alphavirus expression vector for HBc synthesis within 24 h [30,53], which cannot be achieved in HBV-producing cell cultures [32]. According to our protocol, there is a potential for the production of recombinant alphaviruses containing different HBV genotypes and HBc mutant variants, thus providing an opportunity to study the differences in the effects of capsid self-assembly inhibitors on different HBV genotypes and clinical mutants, allowing, to a large extent, individualized drug treatment testing.

The BHK-21 cells transduced with recombinant SFV/HBc virus express the HBc protein which is assembled into capsids [30]. Incubation with a potential capsid assembly inhibitor in cell growth medium followed by native agarose gel electrophoresis of cell lysates and subsequent immunoblot analysis allowed observation of the large-molecule HBc assembly products. By native agarose electrophoresis, it is possible to determine changes in capsid amount and their properties, including mobility in the gel, which depends on the charge in the structure and molar mass, and may indicate changes in the formation of the capsids and their nucleic acid content [37]. The sensitivity of this method by our estimation starts at 10 ng of HBV capsids, and even with a small content of capsids in the lysates, it is possible to detect changes in comparison with untreated control cells. The SFV established model ensures high production of HBc in BHK-21 cells.

In this study, synthesized groups of HAP derivatives and analogues revealed different types of activity on HBc self-assembly. Some derivatives—comp. **1b**, **1e**, and **1i** (Figure 6) showed promising effects by inducing dose-dependent decreases in the quantity of assembled capsids at concentrations less than those needed to affect cell viability. Several synthesized HAPs, e.g., comp. **1d** and **1f**, caused the formation of smears and rather strong signals at the start position in native agarose gel electrophoresis compared to the standard agent Bay 41-4109 (Figure 6). Additionally, comp. **1f**, **1j**, and **1k** revealed similar high molecular mass smear formations (Figure 5). In the case of comp. **1e**, capsid stabilization and accumulation were also detected at 5 μM and 10 μM and a signal smear was observed, which might indicate non-capsid HBc assembly products and/or the formation of protein aggregates. At higher concentrations (up to 20 μM and 50 μM), the capsids were almost non-detectable.

Interestingly, we observed the accumulation and possible aggregation of capsid proteins under treatment with comp. **1d** and **1f**. On the other hand, we did not observe Bay 41-4109 dependent capsid aggregates, the morphology of which was described in the literature as plates or cylinders by in vitro capsid assembly experiments with *E. coli* derived HBc [10]. Although capsid aggregates for Bay 41-4109 were not detected in BHK-21 cells, this does not mean that they do not occur. It was shown previously that non-assembled and defective HBc proteins in BHK-21 cells are rapidly degraded through the proteasomal pathway [53], this factor has no influence on in vitro capsid assembly experiments. The rate of degradation of the HBc aggregates may depend also on the amount and rate of aggregate formation or the nature of the aggregate structures themselves, suggesting different misdirecting assembly mechanisms compared with the reference compound Bay 41-4109.

To evaluate the intracellular localization of the HBc polymer structures induced by comp. **1d**, we performed cell immunostaining analysis. Monoclonal anti-HBc antibodies used for immunostaining recognized both HBc and its dimers as well as assembled capsids [54]. In HBc-producing BHK-21 cells, the HBc protein was evenly distributed in both the cytoplasm and the nucleus (Figure 10C). In contrast, cells treated with comp. **1d** showed granular appearance of HBc and/or capsids only in the cytoplasm. Moreover, an increase in the amount of HBc with increasing compound concentration was observed, indicating the accumulation of HBc-generated polymer structures in cells and disturbed cytoplasmic and nuclear transport (Figure 10A,B).

High efficiency of mammalian HBc production in SFV system allowed us to investigate the capsid-like structures by electron microscopy. Capsid-like structures isolated from transduced BHK-21 cells after treatment with comp. **1d** had different diameters, irregular forms and larger opened compositions as observed by electron microscopy (Figure 11). In studies with Bay 41-4109, *E. coli* produced truncated HBc protein formed tubular structures ranging from 0.6 μm to 1.5 μm in length and from 30 nm to 50 nm in diameter [10]. Interestingly, the Bay 41-4109 treatment of HBc expressing mammalian cells (Huh-7, human hepatoma cell line) led to core particle aggregation, as shown by electron microscopy of cell ultrathin sections [55].

Two of the studied comp., **1d** and **1e**, had significant antiviral effects, showing up to a 93.5% decrease in the amount of HBV virion release compared with control HepG2.2.15 cells without treatment (Figure 8); furthermore, a series of compound dilutions and specific inhibitory concentrations in which HBV replication was inhibited by 50% (EC_50_ values) were determined (Figure 9). Both comp. **1d** and **1e** showed close EC_50_ values of 6.24 and 9.16 μM, respectively (Figure 9C). For Bay 41-4109, the EC_50_ was calculated to be approximately 0.35 μM. The EC_50_ values for Bay 41-4109 in the literature in HepG2.2.15 cells range from 0.085 to 0.5 μM [51,56]. In contrast, the toxicity of comp. **1d** was lower comparing to Bay 41-4109: the calculated TC_50_ for comp. **1d** was 175 μM versus 58 μM for Bay 41-4109. The selectivity index (SI) used to compare the drugs shows the relationship between toxicity and efficacy (TC_50_/EC_50_). Of the compounds studied, the highest SI value was for Bay 41-4109 (Figure 9C). Despite the much more favourable SI of Bay 41-4109, the estimated effective and toxic concentrations of new promising comp. **1d** and **1e** in HepG2.2.15 cells were approximately in the range of some currently approved HBV therapeutic drugs, also taking into account that the studied compounds were used as racemic mixtures. For example, the published in vitro antiviral activity of lamivudine shows EC_50_ values ranging between 0.01 μM and 3.3 μM and for adefovir, between 0.2 μM and 6.3 μM, depending on the cell model system and protocol used [57].

The HAP compounds generated and assessed in this study potentially can be used for investigation of HBc assembly and HBV envelopment using extensive array of computational methods together with the efficient SFV based biological analysis in mammalian cells. The testing approach developed in this study can be used for other viruses, including HIV capsids [58] and herpesvirus capsids [59]. Understanding virus assembly mechanisms would provide a scientific background for the purposeful synthesis of new non-toxic substances with significant antiviral activity and the development of new, promising antiviral compounds for further research and finally for clinics.

## 4. Materials and Methods

### 4.1. Chemicals

The synthesis of HAP derivatives, analogues and the control Bay 41-4109 (comp. **1a**) (see Figure 2, Table 1) are described in detail in the Supplementary data file. Reagents and solvents were purchased from the commercial suppliers Acros Organics (Geel, Belgium), Sigma-Aldrich/Merck KGaA (Darmstadt, Germany) or Alfa Aesar (Lancashire, UK) and used without further purification.

Briefly, HAPs **1a** (Bay 41-4109 rac) and **1b** are known in the literature and were synthesized according to general methods [60,61]. HAPs **1c-1k** were synthesized in a multicomponent (appropriate amidine, aromatic aldehyde, ketoester, or anilide) heterocyclization reaction, according to [62,63,64].

To obtain organic cations **3a-d** and bicyclic dihydropyrimidine **5**, bromination of dihydropyrimidine **1a** was performed with NBS (N-bromosuccinimide) in CCl_4_ solution. Then, acquired bromine derivative **2** [65] was further used in the reactions with the tertiary amines (to form the ammonium compounds) or heated (to perform cyclization), depending on the desired product. HAPs **4a** and **4b** were obtained by aromatization of comp. **1j** and **1k**, respectively, using 2,3-dichloro-5,6-dicyanobenzoquinone (DDQ) as the oxidant. Dihydropyridine **6** (or deaza-HAP) was synthesized via the cyclocondensation reaction of the aminovinylcarbonyl moiety and arylidene carbonyl compound, analogous to Sharma et al. [66]. 1-Alkyl-4-methylpyridin-1-ium 4-methylbenzenesulfonates **7a** and **7b** were synthesized according to Dubur et al. [67].

### 4.2. Molecular Modeling

A homology model of the HBV capsid hexamer subunit was built using SWISS-MODEL [68,69,70,71,72] with the wild-type HBc structure (PDB ID: 6HTX) [29] as the homology template. Three amino acid mutations were introduced in the model (Trp33Gly, Val74Gly, and Ala80Ile) to obtain the sequence identical to the used in the experiments. The structure was prepared for docking using the standard Schrödinger Protein Preparation Wizard software protocol [73]. Protein preparation included adjusting the side chain protonation states at pH 7.0 and energy minimization, allowing heavy atom convergence of up to 0.3 Å, and optimization of the hydrogen bond network. Preparation of the inhibitors was performed using LigPrep at pH 7.2. Induced fit docking (IFD) simulations were performed using the Schrödinger software package release 2018-4 [74,75,76,77]. The cut-off for pose refinement was 30 kcal/mol, and up to 20 poses were retained for each ligand. The ranking of the final docked models was based on the Schrödinger IFD Glide score.

### 4.3. Cell Cultures

BHK-21—Baby hamster kidney cells (ATCC) were maintained in BHK-21 Glasgow modified Eagle’s medium (GMEM) containing 5% fetal bovine serum, 10% tryptose phosphate broth, 2 mM L-glutamine, 100 units/mL penicillin, 100 μg/mL streptomycin, and 20 mM HEPES buffer.

The HepG2.2.15 human hepatoma cell line stably expressing HBV [36] was maintained in Dulbecco’s modified Eagle’s medium containing 10% fetal bovine serum, 400 μg/mL geneticin, 2 mM l-glutamine, and 40 μg/mL gentamycin. Cells were seeded twice a week into collagen-coated flasks. For flask preparation, sterile rat tail collagen solution (Invitrogen; 50 μg/mL in 0.02 M acetic acid) was used according to the manufacturer’s instructions.

### 4.4. HBc Protein Production in BHK-21 Cells

The cytoplasmic expression of the HBc gene driven by exogenously delivered replication-deficient alphavirus was used for high-level production of HBc and HBV capsids in eukaryotic cells. Recombinant pSFV1/HBc virus particles were produced by in vitro RNA transcription from the corresponding recombinant pSFV1 vector plasmid harboring the HBc D1 gene (GenBank Accession No. X02496) and subsequent electroporation together with pSFV1 helper RNA [78] into BHK-21 cells as described in [79,80]. The cell medium containing pSFV1/HBc virus particles was collected and frozen next day after electroporation. The titre of the recombinant pSFV1/HBc virus particles was assessed by immunostaining for the HBc protein with anti-HBc antibodies in infected BHK-21 cells [30]. For infection, BHK-21 cells were incubated with a range of dilutions of recombinant pSFV1/HBc alphavirus particles for one hour in PBS with magnesium and calcium salts (PBS+/+, Gibco). After one hour, cell culture medium was added. The virus titre was calculated as previously described [81].

For HBc expression and capsid assembly analysis, the BHK-21 cells were cultured until they reached 70–80% monolayer confluency, and then cells were infected by incubation with recombinant pSFV1/HBc alphavirus particles at a concentration of 2 × 10^7^ infectious units (iu) per millilitre to achieve almost 100% infection of the cells.

### 4.5. Evaluation of Compound Toxicity

The cytopathic effect of the compounds was estimated by phase contrast microscopy and standard trypan blue staining, and the cells were counted using an automatic cell counter (Invitrogen). BHK-21 and HepG2.2.15 cells were incubated for 24 h with the studied compounds at four serial dilutions (25, 50, 100, 150 and 200 μM) and the cell morphology was controlled under microscope to evaluate cytopathic effects (Table 1, the highest non-toxic concentration).

Additionally, cell viability was determined by tetrazolium salt-based assay using the Vybrant MTT Cell Proliferation kit (Thermo Fisher Scientific, Waltham, MA, USA) or more sensitive CCK-8 kit (Abcam). First, the cell numbers and protocol were optimized for BHK-21 and HepG2.2.15 cells, as indicated in the MTT assay kit or CCK-8 reagent manufacturer’s protocol, and standard curves with OD values were created and evaluated.

In order to evaluate the toxicity of selected HAP compounds in the medium, 1.3 × 10^4^ BHK-21 were seeded per well in 96-well plates and treated with the studied compounds for 24 h at concentrations 6.125; 12.25; 25; 50; 100; and 200 μM. Control cells without HAP compounds and MTT-free cell media were included in the experiments. The MTT test was performed according to the full protocol version (Thermo Fisher Scientific).

In order to calculate the median of the toxic concentration (TC_50_) of the compounds, 1.33 × 10^5^ HepG2.2.15 cells per well were seeded into a 24-well plate. After 24 h, the cells were treated with the test compounds at different concentrations (0, 50, 100, 150, 200 μM) and incubated for 48 h at 37 °C. All experiments were carried out in three identical repeats and the coefficient of variation and standard deviation were calculated from at least two independent experiments.

### 4.6. Evaluation of Compound Effects on HBV Capsid Assembly in BHK-21 Cells

Evidence of HBc assembly was assessed directly in the corresponding BHK-21 cell lysates by native agarose gel electrophoresis followed by capillary transfer to nitrocellulose membrane and standard Western blot analysis (immunoblot), as described elsewhere [82]. Briefly, 70% confluent BHK-21 cells cultured in 24-well plates were infected with 150 μL of recombinant pSFV1/HBc virus stock (2 × 10^7^ iu/mL). After 4 h, the cell culture medium was replaced with inhibitor-containing medium, and the cells were incubated at 37 °C for 24 h. Test compounds were diluted from their 10 mM stock solutions in DMSO to 1 mM in PBS and then added to the cell medium at final concentrations of 1 μM, 5 μM, 10 μM, and 20 μM for dose-dependent effect studies or at the highest non-cytopathic concentration according to the morphological examination of compound toxicity to BHK-21 cells. After 24 h of incubation, the cell medium was removed and the cells were lysed by the addition of 250 μL per well of buffer containing 50 mM Tris-HCl (pH 7.4), 150 mM NaCl, 2 mM EDTA, 1% NP-40 and 0.1% protease inhibitor cocktail (Sigma Aldrich, Darmstadt, Germany). Cell debris and nuclei were removed by centrifugation at 3000× *g* for 5 min.

The protein concentration in lysates was measured using a BCA protein assay kit (Pierce) according to the manufacturer’s protocol, and the lysates were equalized by protein content. Thirty microlitres of the corresponding protein equalized lysates were fractionated by electrophoresis through nondenaturing 1–1.5% agarose gels and transferred via a capillary transfer to a nitrocellulose Hybond C extra (Amersham BioSciences) membrane by blotting with 10× SSC buffer (Thermo Fisher Scientific). HBV capsids were detected by probing the membrane with polyclonal rabbit antibodies against the HBV core protein (Dako, cat. No. B058601) diluted 1:2000. Bound antibodies were revealed by horseradish peroxidase (HRP)-labelled secondary antibodies and visualized with Pierce SuperSignal West Femto Western blotting substrate (Thermo Fisher Scientific).

### 4.7. HBV Virion DNA Isolation from the HepG2.2.15 Cell Culture Supernatant

HepG2.2.15 culture conditions were optimized for HBV production, which was controlled by measuring the levels of secreted HBsAg protein using the ETI-AB-AUK PLUS ELISA kit (anti-HBs; DiaSorin, Saluggia, Italy) according to the manufacturer’s instructions.

HepG2.2.15 cells were cultured in the presence of the studied HAP compounds in 24-well plates for six days. For the experiment, 1.33 × 10^5^ cells were seeded per well into a collagen-coated 24-well plate. After 24 h, the test compounds were diluted from a 10 mM stock solution in DMSO to a 1 mM concentration in PBS and then added to 0.5 mL 2% fetal bovine serum-containing media at final concentrations of 5 μM, 7.5 μM, 10 μM, 15 μM, and 20 μM, and used for HepG2.2.15 cell cultivation. After 72 h, the medium was replaced with the same compound containing freshly prepared medium, and the cells were incubated for additional 72 h. Then, the virus-containing cell culture media was collected, and DNA was isolated immediately after collection of the medium. Viral DNA samples were prepared by the inorganic extraction method [83]. The cells and debris were removed by centrifugation at 2000× *g* for 10 min followed by centrifugation of the supernatant at 13,000× *g* for 10 min. Five units of DNase-I (Thermo Fisher Scientific) per 1 mL were added to the supernatant, and the sample was incubated for 20 min at 37 °C. DNase was inactivated by heating the sample for 10 min at 75 °C. To release the HBV DNA from the capsids, the samples were treated with 1 mg/mL proteinase K with 0.66% SDS for 1 h at 56 °C. After one hour, 10^7^ copies of the pEGFP-C1 (Clontech) plasmid standard were added to the sample, which served as the DNA isolation control. The sample was vortexed and incubated for another 1 h at 56 °C. To remove proteins, ammonium acetate was added to the sample to a final concentration of 3.4 M, and the sample was centrifuged at 11,000× *g* for 30 min. After the addition of sodium acetate to a final concentration of 0.3 M and 2.5 μg of glycogen, the DNA was precipitated from the supernatant with 2.5 volumes of 96% ethanol. Samples were incubated at −20 °C overnight or for at least four hours and then centrifuged at 13,000× *g* for 30 min. After washing with 70% ethanol twice, the dried samples were dissolved in 100 μL of sterile distilled water.

### 4.8. HBV Virion DNA Quantification by Real Time PCR

For absolute HBV DNA quantification, the DNA standards were prepared from the plasmid pEGFP-C1 (Clontech)—EGFP gene-containing plasmid to control the DNA isolation steps, and pSFV1 vector harbouring the complete HBV genome from the pHB320 plasmid, genotype D, subtype “ayw” (GenBank Accession No. X02496), as an HBV control [80,82]. Briefly, linearized and purified from agarose gel plasmids were diluted in distilled nuclease-free water containing 0.3 mg/mL lambda phage DNA so that the final 5 μL standard would contain 1 × 10^7^, 1 × 10^6^, 5 × 10^5^, 1 × 10^5^, 5 × 10^4^, 1 × 10^4^, 5 × 10^3^, or 1 × 10^3^ copies. The prepared standards were verified by real-time PCR and stored directly in PCR tubes at −20 °C or −70 °C for longer periods of time. For the amplification of the EGFP 174 bp gene fragment, the following primers were used: position 748–776, GFP Forward 5′- AAG TTC ATC TGC ACC ACC G-3′; position 921–901 GFP Reverse 5′- TCC TTG AAG AAG ATG GTG CG-3′. The primers used for amplification of the 84 bp HBc gene fragment were: HBV C forward 5′-CGG AGA CTA CTG TTG TTA GAC-3′ positions in HBV genome 2332–2353 nt and HBV C reverse 5′-GCG GCG ATT GAG ACC TTC-3′ positions in HBV genome 2416–2399 nt (GenBank Accession No. X02496).

Two parallel reactions were carried out on each sample tested by quantifying both HBV DNA and EGFP DNA using SYBR Green PCR Master Mix (Thermo Scientific) in MiniOpticon Real-Time PCR Detection System (Bio-Rad), according to the manufacturer’s instructions. The quality of HBV DNA extraction was assessed by an internal pEGFP standard with a known number of copies added to each sample prior to DNA extraction (see section HBV DNA isolation from HepG2.2.15 cell culture). Samples with EGFP DNA variation values that exceeded 20% were excluded from analysis. All samples were analysed in three replicates.

To evaluate the effects of the inhibitors, the amount of HBV virion DNA isolated from the cellular culture after incubation of the cells with different studied compounds was compared. The DNA isolated from HepG2.2.15 cells that were not treated with the studied compounds was used as a control. Using the obtained data from each sample, a trend curve with the closest linearity R^2^ = 1 value was created, and the effective concentration EC50 value of the inhibitor was calculated (50% of HBV DNA reduction).

### 4.9. HBc Protein Detection in BHK-21 Cells by Immunostaining

To determine HBc production in cells as well as to assess the intracellular localization of the capsids and HBc protein in BHK-21 cells, indirect immunofluorescence analysis was performed. BHK-21 cells were grown on coverslips in 24-well plates to 70% confluency. After infection with pSFV1/HBc and incubation with the studied compounds, the cells were washed twice with PBS and air dried. Cells were fixed with cold (−20 °C) methanol, washed twice with PBS, and then blocked for 15 min at 37 °C (0.5% BSA, 0.5% fetal bovine serum in PBS). Cells were incubated with primary anti-HBc antibody mouse monoclonal antibody C1-5 [54] in blocking solution for 1 h at 37 °C. After incubation, the cells were washed three times with PBS and the secondary Alexa Fluor-488 labelled anti-mouse antibody in blocking solution was added followed by incubation for 30 min at 37 °C. Then, the cells were washed three times with PBS, incubated with the DNA stain reagent DAPI (1 mg/mL, dilution 1:10,000 in PBS) for 10 min at room temperature. Cells were mounted into Mowiol mounting medium with 2.5% DABCO (Sigma Aldrich, Darmstadt, Germany), left at room temperature in the dark overnight and examined for DAPI and HBc staining by confocal fluorescence microscopy (Leica TCS Sp2 SE) as previously described [30].

### 4.10. HBV Capsid Isolation from BHK-21 Cells and Evaluation by Electron Microscopy

BHK-21 cells were cultured in two 75 cm^2^ cell culture flasks until they reached 80% monolayer confluency. Cells were infected by incubation with recombinant pSFV1/HBc alphavirus (10^7^ iu per mL) for one hour in serum-free cell media. After one hour, BHK-21 cell culture medium was added. Three hours post-infection, 50 μM comp. **1d** was added, and the cells were further incubated for 24 h at 37 °C. Cells without compound were used as a control. The cells were trypsinized, washed with PBS+/+, collected in a 15 mL tube, and lysed for 48 h on ice with two milliliters of lysis buffer, containing 1% NP-40 with 0.1% proteinase inhibitor cocktail (Sigma). Cell lysates were centrifuged for 5 min at 3000× *g* and 15 min at 10,000× *g* to remove NP-40 insoluble cell debris. Capsids from the lysates were pelleted by ultracentrifugation at 111,000× *g* at 4 °C for four hours through a 20% sucrose cushion in 0.5 M NaCl/PBS solution using SW40 Ti rotor. The precipitate was dissolved in 10 μL of 0.5 M NaCl solution in PBS, transferred to low-binding protein tubes and analyzed by electron microscopy (EM). The EM visualization was performed with uranyl acetate negative staining. First, 5 µL of the sample was absorbed on carbon formvar-coated 300 Mesh Copper grids (Agar Scientific, Stansted, UK; 2 grids per sample were prepared) and incubated for 3 min. The grids were then washed with 1 mM ethylenediaminetetraacetic acid (EDTA) and negatively stained with 0.5% uranyl acetate aqueous solution. The grids were analyzed with a JEM-1230 electron microscope (JEOL, Tokyo, Japan) at an accelerating voltage of 100 kV.

### 4.11. Statistical Analysis

The percent inhibition at each compound concentration was calculated by normalization of the signals to the signals from non-treated HepG2.2.15 cells. In dose–response studies, the compound concentrations were log transformed, and inhibition curves were generated by nonlinear fitting using Microsoft Excel software, from which the EC_50_ (effective concentration) and TC_50_ (toxic concentration) values were determined. Data points and error bars represent the means and standard deviations from at least two independent experiments, respectively. Comparisons between data groups were performed using the unpaired t test, with *p* values < 0.05 being considered statistically significant.

## 5. Conclusions

New anti-HBV HAP compounds of the CAM–A class with reduced cytotoxicity were designed and synthesized. Some compounds, such as **1d**, **1f**, **1j**, **1k**, and **5**, induce the formation of incorrectly formed capsids that accumulate in the cell compared to those initiated by the reference HAP Bay 41-4109.

Two of the compounds studied, **1d** and **1e**, have a significant antiviral effect and a favourable toxicity profile that allows these compounds to be considered promising leads for further research as drug candidates.

The recombinant SFV vector-based expression of the full-length HBc protein represents an excellent model for the evaluation of new anti-HBV CAM-A class compounds in its natural mammalian environment. Application of SFV-based approach led to highly efficient HBc expression, which allows exploration of the effects of studied compounds on capsid assembly as well as a direct evaluation of the drug impact on their target under natural conditions and simultaneous toxicity assessment, avoiding the full cycle of virus replication and subsequent safety concerns.

## Figures and Tables

**Table 1:** Compound structures, their toxicity, and effects on HBV capsid assembly. (**A**)—compounds **1 a-k** and **3 a-d**; (**B**)—compounds **4 a**, **b**, **5**, **6**, and **7 a**, **b**.

**(A)**						
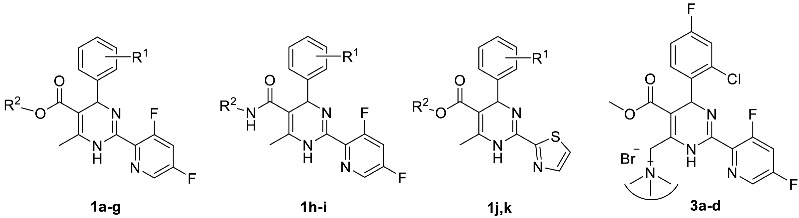						
**Nr.**	**R^1^**	**R^2^**	**N^+^**	**The Highest Non-Toxic Concentration, μM**	**Effect on Capsid Assembly, Starting Concentration**	
**Bay 41-4109 (1a)**	2-Cl, 4-F	Me		100	Dose dependent capsid signal decrease, 1 μM	
**1b**	2-Cl, 4-F	H		50	Dose dependent capsid signal decrease, 10 μM	
**1c**	4-F	Et		<25	Capsid signal decrease, 5 μM	
**1d**	2-Cl, 4-F	CH_2_CH_2_OEt		200	HBc accumulation, aggregates/or incorrect capsid structures, 5 μM	
**1e**	2-Cl, 4-F	CH_2_CH_2_O-Pr-i		100	Dose dependent capsid signal decrease, 10 μM	
**1f**	2-Cl, 4-F	CH_2_CH_2_OPr		100	HBc accumulation, aggregates/or incorrect capsid structures, 10 μM	
**1g**	2-OCHF_2_	CH_2_CH_2_OPr		50	Insignificant capsid amount increases	
**1h**	2-Cl, 4-F	2-pyridyl		150	Dose dependent capsid signal decrease, 20 μM	
**1i**	2-Cl, 4-F	*o*-methoxyphenyl		50	Dose dependent capsid signal decrease, 20 μM	
**1j**	4-F	Et		<25	HBc accumulation, aggregates/or incorrect capsid structures, 5 μM	
**1k**	H	Et		25	Dose dependent HBc accumulation, aggregates/or incorrect capsid structures, 1 μM	
**3a**			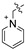	200	No effect	
**3b**			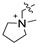	200	No effect	
**3c**			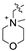	150	No effect	
**3d**			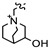	100	Capsid signal decrease, possible changes in NS packaging	
**(B)**						
	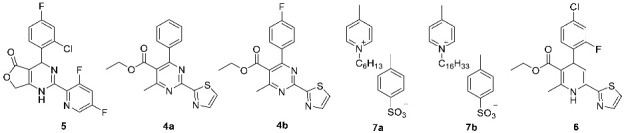					
**The highest non-toxic concentration, μM**	200	25	200	50	<25	150
**Effect to capsid assembly, starting concentration, μM**	HBc accumulation, aggregates/or incorrect capsid structures, 50 μM	No effect	No effect	No effect	No effect	No effect

## Data Availability

Data is contained within the article and supplementary materials.

## Supplementary Materials

The following supporting information can be downloaded at: https://www.mdpi.com/article/10.3390/ph15070773/s1, methods for the synthesis of compounds **1a-k**, **3a-d**, **5**, and **7a,b**.
