# Supplementary material for: Design and Synthesis of Hepatitis B Virus (HBV) Capsid Assembly Modulators and Evaluation of Their Activity in Mammalian Cell Model
Source: Pharmaceuticals (Basel). 2022 Jun 22;15(7):773. doi: 10.3390/ph15070773 (PMC9317397; doi:10.3390/ph15070773)
Supplement: Supplementary file 1 [file pharmaceuticals-15-00773-s001.zip › pharmaceuticals-1725344-supplementary.pdf]

*Type of the Paper: Article*

# Design and synthesis of hepatitis B virus (HBV) capsid assembly modulators and evaluation of their activity in mammalian cell model

**Karina Spunde[1*], Brigita Vigante[2*], Unda Nelda Dubova[1], Anda Sipola[2], Irena Timofejeva[1], Anna Zajakina[1], Juris Jansons[1], Aiva Plotniece[2], Karlis Pajuste[2], Arkadij Sobolev[2], Ruslan Muhamadejev[2], Kristaps Jaudzems[2], Gunars Duburs[2], Tatjana Kozlovska[1].**

[1] Latvian Biomedical Research and Study Centre, LV-1067 Riga, Latvia; spunde.carina@gmail.com (K.S.); undadubova@gmail.com (U.N.D.); Irena@biomed.lu.lv (I.T.); anna@biomed.lu.lv (A.Z.); jansons@biomed.lu.lv (J.J); tatyana@biomed.lu.lv (T.K.)

[2] Latvian Institute of Organic Synthesis, LV-1006 Riga, Latvia; vigante@osi.lv (B.V.); anda@osi.lv (A.S.); aiva@osi.lv (A.P.); kpajuste@osi.lv (K.P.); arkady@osi.lv (A.So.); muhamadejev@osi.lv (R.M.); kristaps.jaudzems@osi.lv (K.J.); gduburs@osi.lv (G.D.)

* Correspondence: Karina Spunde[1*] *e-mail:* spunde.carina@gmail.com; Brigita Vigante[2*] *e-mail:* vigante@osi.lv

# Supplementary material

## Synthesis and characterization of HAP compounds

## General

Reagents and solvents were purchased from the commercial suppliers Acros Organics (Geel, Belgium), Sigma-Aldrich/Merck KGaA (Darmstadt, Germany) or Alfa Aesar (Lancashire, UK) and used without further purification. Thin-layer chromatography (TLC) was performed on silica gel 60 F254 aluminium sheets 20 cm × 20 cm (Merck KGaA, Darmstadt, Germany) and visualized by UV (254 nm) fluorescence. ZEOCHEM silica gel (ZEOprep 60/35–70 microns: SI23501) or SNAP KP-C18-HS, 50μm Irregular silica Flash Cartridges (Biotage, Uppsala Sweden) were used for flash chromatography. Melting points were recorded on an OptiMelt digital melting point apparatus (Stanford Research Systems, Sunnyvale, CA, USA) and are uncorrected. The $^{1}H$, $^{13}C$ and $^{19}F$ NMR spectra were recorded on a Bruker Avance Neo 400 MHz (Bruker Biospin Gmbh, Rheinstetten, Germany) equipment at 400.0, 100.58 and 376.0 MHz, respectively, at 298 K in $CDCl_3$ or $CD_3OD$. The chemical shifts of the hydrogen and carbon atoms are presented in parts per million (ppm) and referred to the residual signals of the non-deuterated $CDCl_3$ (δ: 7.26) or $CD_3OD$ (δ: 4.87 and 3.31) solvent for $^{1}H$-NMR spectra and $CDCl_3$ (δ: 77.0) or $CD_3OD$ (δ: 49.0) solvent for $^{13}C$-NMR, respectively. Coupling constants, *J*, were reported in hertz (Hz). Elemental analyses were determined on an Elemental Combustion System ECS 4010 (Costech International S.p.A., Milano, Italy) and high-resolution mass spectra (HRMS) were determined on an Acquity UPLC H-Class system (Waters, Milford, MA, USA) connected to a Waters Synapt GII Q-ToF operating in the ESI positive or negative ion mode on a Waters Acquity UPLC® BEH C18 column (1.7 μm, 2.1 × 50 mm, using gradient elution with acetonitrile (0.01% formic acid) in water (0.01% formic acid) at Laboratory of Chromatography of Latvian Institute of Organic Synthesis

## Synthesis of HAP compounds:

### General method for the synthesis of substituted heterylaryldihydropyrimidines 1a-k.

To a mixture of appropriate amidine (0.005 mol; 1eq), aromatic aldehyde (0.005 mol, 1.00 eq) and β-ketoester or *N*-substituted carboxamide (0.005 mol, 1.00 eq) in *i*-PrOH (15 mL) was added anhydrous NaOAc (1.00 eq) and the reaction mixture was stirred for 5 h under argon atmosphere at 90°C. Then the reaction mixture was evaporated and the remaining residue was dissolved in EtOAc (~10 mL) and washed with 1M HCl (3×5 mL). HCl extracts were combined and washed with $Et_2O$ (3×3 mL). Then 20% NaOH solution in water was added to HCl extract till precipitate appear. Then suspension was extracted with EtOAc (3×10 mL), organic layers were combined and washed with brine, dried with anhydrous $Na_2SO_4$, filtered and evaporated. Crude product was purified with flash chromatography on silica gel with EtOAc (10-50%) in petroleum ether (bp 40-60°C). Product was recrystallized from $MeOH/H_2O$ to give pure products **1a-l**.

Synthesis and characterization of HAPs derivatives **1a** (Bay 41-4109-rac) and **1b** have been reported in the literature [1, 2]. $^{1}H$ and $^{13}C$ NMR spectral data of the resynthesized samples were in agreement with those reported in literature.

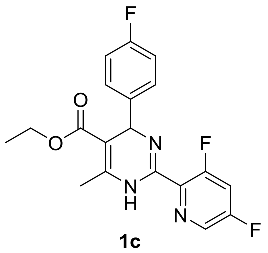

**1c**

**Ethyl 2-(3,5-difluoropyridin-2-yl)-4-(4-fluorophenyl)-6-methyl-1,4-dihydropyrimidine-5-carboxylate (1c):**

Yield: 601 mg, 32%

Melting point (MeOH/$H_2O$): 126-127°C

Anal. calcd for $C_{19}H_{16}F_3N_3O_2$: C, 60.80; H, 4.30; N, 11.20; found: C, 60.88; H, 4.35; N, 11.12.

$^1$H NMR (400 MHz, $CDCl_3$, δ): 8.30 (d, *J* = 2.3 Hz, 1H), 8.25 (s, 1H), 7.42 – 7.36 (m, 2H), 7.33 (ddd, *J* = 10.3, 8.0, 2.3 Hz, 1H), 6.99 – 6.92 (m, 2H), 5.95 (s, 1H), 4.13 (q, *J* = 7.1 Hz, 2H), 2.47 (s, 3H), 1.22 (t, *J* = 7.1 Hz, 3H) ppm.

$^{13}$C NMR (100 MHz, $CDCl_3$, δ): 166.9, 162.1 (d, *J* = 244.7 Hz), 160.2 (dd, *J* = 266.0, 6.0 Hz), 158.8 (dd, *J* = 278.0, 6.0 Hz), 145.6 (d, *J* = 10.3 Hz), 144.9, 140.7 (d, *J* = 3.2 Hz), 134.5 – 134.3 (m), 132.5 (dd, *J* = 24.3, 4.7 Hz), 129.0 (d, *J* = 8.1 Hz), 115.2 (d, *J* = 21.2 Hz), 114.1 (t, *J* = 21.8 Hz), 99.4, 60.0, 58.8, 19.1, 14.4 ppm.

$^{19}$F NMR (376 MHz, $CDCl_3$, δ): -110.1 (t, *J* = 10.5 Hz), -115.9 – -116.0 (m), -119.2 (dd, *J* = 10.8, 8.0 Hz) ppm.

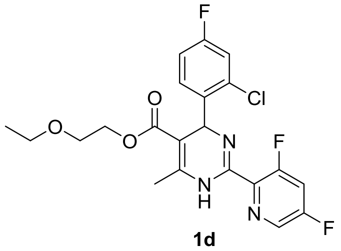

**1d**

**2-Ethoxyethyl 4-(2-chloro-4-fluorophenyl)-2-(3,5-difluoropyridin-2-yl)-6-methyl-1,4-dihydropyrimidine-5-carboxylate (1d):**

Yield: 635 mg, 28%

Melting point (MeOH/$H_2O$): 143-144°C

Anal. calcd for $C_{21}H_{19}ClF_3N_3O_3$: C, 55.58; H, 4.22; N, 9.26; found: C, 55.36; H, 4.21; N, 9.08.

$^1$H NMR (400 MHz, $CDCl_3$, δ): 8.27 (d, *J* = 2.3 Hz, 1H), 8.22 (s, 1H), 7.34 – 7.27 (m, 2H), 7.13 (dd, *J* = 8.7, 2.6 Hz, 1H), 6.90 (td, *J* = 8.3, 2.6 Hz, 1H), 6.33 (s, 1H), 4.16 – 4.12 (m, 2H), 3.54-3.49 (m, 2H), 3.44 – 3.35 (m, 2H), 2.54 (s, 3H), 1.13 (t, *J* = 7.0 Hz, 3H) ppm.

$^{13}$C NMR (100 MHz, $CDCl_3$, δ): 166.4, 161.6 (d, *J* = 248.7 Hz), 160.2 (dd, *J* = 265.2, 6.3 Hz), 158.8 (dd, *J* = 279.2, 6.3 Hz), 146.3, 144.9 (d, *J* = 10.3 Hz), 138.0 (d, *J* = 3.5 Hz), 134.4 – 134.2 (m), 134.2 (d, *J* = 10.3 Hz), 132.4 (dd, *J* = 23.7, 4.9 Hz), 130.4 (d, *J* = 8.7 Hz), 117.2 (d, *J* = 24.6 Hz), 114.2 (d, *J* = 20.8 Hz), 113.8 (t, *J* = 22.5 Hz), 97.5, 68.5, 66.7, 63.5, 56.6, 19.0, 15.3 ppm.

$^{19}$F NMR (376 MHz, $CDCl_3$, δ): -109.4 (t, *J* = 10.5 Hz), -113.8 – -113.9 (m), -119.2 (dd, *J* = 10.9, 8.1 Hz) ppm.

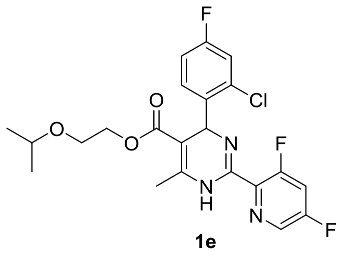

1e

**2-Isopropoxyethyl 4-(2-chloro-4-fluorophenyl)-2-(3,5-difluoropyridin-2-yl)-6-methyl-1,4-dihydropyrimidine-5-carboxylate (1e):**

Yield: 866 mg, 37%
Melting point (MeOH/$H_2O$): 156-157°C
Anal. calcd for $C_{22}H_{21}ClF_3N_3O_3$: C, 56.48; H, 4.52; N, 8.98; found: C, 56.24; H, 4.45; N, 8.93.
$^1H$ NMR (400 MHz, $CDCl_3$, δ): 8.27 (d, *J* = 2.2 Hz, 1H), 8.26 – 8.04 (br. s, 1H), 7.34 – 7.28 (m, 2H), 7.13 (dd, *J* = 8.6, 2.6 Hz, 1H), 6.90 (td, *J* = 8.4, 2.6 Hz, 1H), 6.29 (s, 1H), 4.14 (dd, *J* = 5.6, 4.4 Hz, 2H), 3.54 – 3.48 (m, 3H), 2.55 (s, 3H), 1.09 (dd, *J* = 10.6, 6.1 Hz, 6H) ppm.
$^{19}F$ NMR (376 MHz, $CDCl_3$, δ): -108.9 – -109.8 (m), -113.4 – -114.3 (m), -118.7 – -119.5 (m) ppm.

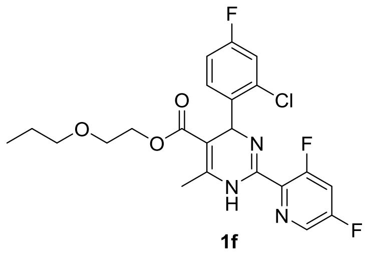

1f

**2-Propoxyethyl 4-(2-chloro-4-fluorophenyl)-2-(3,5-difluoropyridin-2-yl)-6-methyl-1,4-dihydropyrimidine-5-carboxylate (1f):**

Yield: 983 mg, 42%
Melting point (MeOH/$H_2O$): 149-150°C
Anal. calcd for $C_{22}H_{21}ClF_3N_3O_3$: C, 56.48; H, 4.52; N, 8.98; found: C, 56.24; H, 4.45; N, 8.93.
$^1H$ NMR (400 MHz, $CDCl_3$, δ): 8.37 – 8.12 (m, 2H), 7.36 – 7.23 (m, 2H), 7.12 (dd, *J* = 8.6, 2.6 Hz, 1H), 6.89 (td, *J* = 8.3, 2.6 Hz, 1H), 6.29 (s, 1H), 4.20 – 4.10 (m, 2H), 3.58 – 3.46 (m, 2H), 3.37 – 3.23 (m, 2H), 2.54 (s, 3H), 1.56 – 1.44 (m, 2H), 0.87 (t, *J* = 7.4 Hz, 3H) ppm.
$^{13}C$ NMR (100 MHz, $CDCl_3$, δ): 166.4, 161.7 (d, *J* = 248.7 Hz), 160.2 (dd, *J* = 266.2, 5.5 Hz), 158.8 (dd, *J* = 279.6, 5.5 Hz), 146.6, 144.9, 137.8 (d, *J* = 3.2 Hz), 134.3 (d, *J* = 9.2 Hz), 134.1 – 133.6 (m), 132.4 (d, *J* = 23.8 Hz), 130.5 (d, *J* = 8.9 Hz), 117.2 (d, *J* = 24.5 Hz), 114.3 (d, *J* = 20.7 Hz), 114.0 (t, *J* = 22.8 Hz), 97.6, 73.0, 68.6, 63.4, 22.9, 19.2, 10.6 ppm.
$^{19}F$ NMR (376 MHz, $CDCl_3$, δ): -109.37 (m), -113.75 (m), -119.07 (m) ppm.

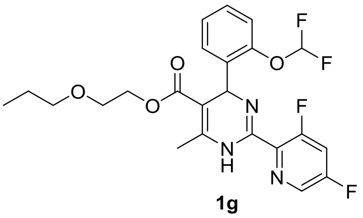


**1g**

**2-Propoxyethyl 4-(2-methoxydifluorophenyl)-2-(3,5-difluoropyridin-2-yl)-6-methyl-1,4-dihydropyrimidine-5-carboxylate (1g):**

Yield: 990 mg, 41%
Melting point (MeOH/$H_2O$): 164-165°C
Anal. calcd for $C_{23}H_{23}F_4N_3O_4$: C, 57.38; H, 4.82; N, 8.73; found: C, 57.09; H, 4.4.72; N, 8.48.
$^1$H NMR (400 MHz, $CDCl_3$, δ): 8.25 (d, *J*=2.2 Hz, 2H), 7.38-7.10 (m, 5H), 6.86 (AB-system, 1H, *J* = 71.4 Hz), 6.28 (s, 1H), 4.13 (t, *J*=4.9 Hz, 2H), 3.55-3.40 (m, 2H), 3.27 (td, *J*=6.7, 4.2 Hz, 2H), 2.54 (s, 3H), 1.50 (q, *J*=7.1 Hz, 2H), 0.86 (t, *J*=7.4 Hz, 3H) ppm.

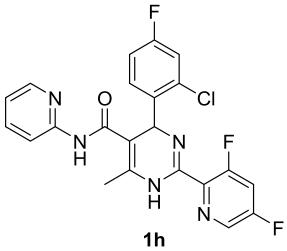


**1h**

**4-(2-Chloro-4-fluorophenyl)-2-(3,5-difluoropyridin-2-yl)-6-methyl-N-(pyridin-2-yl)-1,4-dihydropyrimidine-5-carboxamide (1h):**

Yield: 990 mg, 27%
Melting point (MeOH/$H_2O$): 152-153°C
Anal. calcd for $C_{22}H_{15}ClF_3N_5O$: C, 57.71; H, 3.30; N, 15.30; found: C, 57.34; H, 3.46; N, 15.11.
$^1$H NMR (400 MHz, $CDCl_3$, δ): 8.29 (d, *J* = 2.3 Hz, 1H), 8.22 (d, *J* = 4.4 Hz, 2H), 8.13 (d, *J* = 8.4 Hz, 1H), 7.72 (s, 1H), 7.67 – 7.60 (m, 1H), 7.43 (dd, *J* = 8.7, 6.0 Hz, 1H), 7.31 (ddd, *J* = 10.2, 8.0, 2.3 Hz, 1H), 7.20 – 7.13 (m, 1H), 6.96 (ddd, *J* = 11.0, 6.5, 2.2 Hz, 2H), 6.29 (s, 1H), 2.49 (s, 3H) ppm.
$^{13}$C NMR (100 MHz, $CDCl_3$, δ): 165.4, 160.3 (dd, *J* = 265.9, 5.1 Hz), 162.1 (d, *J* = 249.1 Hz), 158.8 (dd, *J* = 282.4, 5.1 Hz), 151.6, 148.1, 145.9 (d, *J* = 9.5 Hz), 141.9, 138.2, 136.2 (d, *J* = 2.4 Hz), 134.3 – 134.0 (m), 132.6 (dd, *J* = 23.5, 4.3 Hz), 130.6 (d, *J* = 8.9 Hz), 119.7, 117.8 (d, *J* = 24.9 Hz), 114.8 (d, *J* = 20.8 Hz), 114.3, 114.1 (t, *J* = 20.8 Hz), 101.3, 57.2, 18.7 ppm.
$^{19}$F NMR (376 MHz, $CDCl_3$, δ): -109.5 (t, *J* = 10.1 Hz), -112.2 – -112.4 (m), -118.9 (t, *J* = 9.9 Hz) ppm.

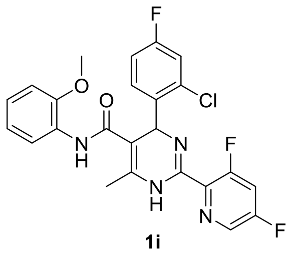

1i

**4-(2-Chloro-4-fluorophenyl)-2-(3,5-difluoropyridin-2-yl)-*N*-(2-methoxyphenyl)-6-methyl-1,4-dihydropyrimidine-5-carboxamide (1i):**

Yield: 560 mg, 23%
Melting point (MeOH/$H_2O$): 149-150°C
Anal. calcd for $C_{24}H_{18}ClF_3N_4O_2$: C, 59.21; H, 3.73; N, 11.51; found: C, 58.74; H, 3.76; N, 11.20.
$^1$H NMR (400 MHz, $CDCl_3$, δ): 8.33 (dd, *J* = 7.9, 1.6 Hz, 1H), 8.28 (d, *J* = 2.3 Hz, 1H), 8.20 (s, 1H), 7.64 (s, 1H), 7.46 (dd, *J* = 8.6, 6.1 Hz, 1H), 7.29 (ddd, *J* = 10.3, 8.1, 2.3 Hz, 1H), 7.22 (dd, *J* = 8.6, 2.6 Hz, 1H), 7.01 – 6.94 (m, 2H), 6.91 (td, *J* = 7.9, 1.6 Hz, 1H), 6.78 (dd, *J* = 7.9, 1.6 Hz, 1H), 6.29 (s, 1H), 3.71 (s, 3H), 2.57 (s, 3H) ppm.
$^{13}$C NMR (100 MHz, $CDCl_3$, δ): 164.6, 162.1 (d, *J* = 249.9 Hz), 160.2 (dd, *J* = 279.6, 6.2 Hz), 158.8 (dd, *J* = 266.7, 6.2 Hz), 148.0, 145.7 (d, *J* = 10.3 Hz), 142.9, 135.8 (d, *J* = 3.4 Hz), 134.5 (d, *J* = 10.4 Hz), 134.3 – 134.0 (m), 132.4 (dd, *J* = 24.0, 5.0 Hz), 131.0 (d, *J* = 9.1 Hz), 128.1, 123.5, 121.1, 119.7, 117.5 (d, *J* = 24.5 Hz), 114.8 (d, *J* = 20.9 Hz), 114.0 (t, *J* = 22.0 Hz), 109.9, 100.8, 57.1, 55.6, 18.7 ppm.
$^{19}$F NMR (376 MHz, cdcl3) δ -109.6 (t, *J* = 10.3 Hz), -112.3 – -112.4 (m), -119.1 (dd, *J* = 10.9, 8.1 Hz) ppm.

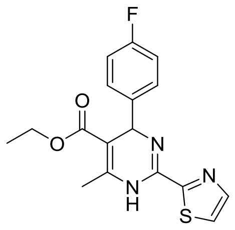

1j

**Ethyl 4-(4-fluorophenyl)-6-methyl-2-(thiazol-2-yl)-1,4-dihydropyrimidine-5-carboxylate (1j):**

Yield: 1.07 g, 44%
Melting point (MeOH/$H_2O$): 114-115°C
Anal. calcd for $C_{17}H_{16}FN_3O_2S$: C, 59.12; H, 4.67; N, 12.17; S, 9.28; found: C, 59.28; H, 4.71; N, 12.17; S, 9.25.
$^1$H NMR (400 MHz, $CDCl_3$, δ): 7.86 – 7.83 (br. s, 1H), 7.82 (d, *J* = 3.2 Hz, 1H), 7.46 (d, *J* = 3.2 Hz, 1H), 7.39 – 7.33 (m, 2H), 7.00 – 6.93 (m, 2H), 5.80 (s, 1H), 4.12 (q, *J* = 7.1 Hz, 2H), 2.46 (s, 3H), 1.21 (t, *J* = 7.1 Hz, 3H) ppm.
$^{13}$C NMR (100 MHz, $CDCl_3$, δ): 166.7, 162.9, 162.2 (d, *J* = 245.0 Hz), 144.5, 144.3, 143.1, 140.7 (d, *J* = 3.1 Hz), 129.1 (d, *J* = 8.0 Hz), 123.3, 115.3 (d, *J* = 21.3 Hz), 100.5, 60.0, 58.8, 19.1, 14.4 ppm.
$^{19}$F NMR (376 MHz, $CDCl_3$, δ): -115.6 – -115.7 (m) ppm.

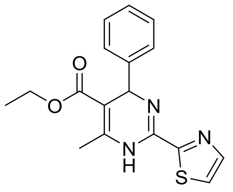

**1k**

**Ethyl 6-methyl-4-phenyl-2-(thiazol-2-yl)-1,4-dihydropyrimidine-5-carboxylate (1k):**

Yield: 688 mg, 42%

Melting point (MeOH/$H_2O$): 129-130°C

Anal. calcd for $C_{17}H_{17}N_3O_2S$: C, 62.36; H, 5.23; N, 12.83; S, 9.79; found: C, 62.35; H, 5.21; N, 12.87; S, 9.68.

$^1H$ NMR (400 MHz, $CDCl_3$, δ): 7.83 – 7.78 (br. s, 1H), 7.79 (d, *J* = 3.1 Hz, 1H), 7.41 (d, *J* = 3.1 Hz, 1H), 7.39 – 7.35 (m, 2H), 7.29 – 7.16 (m, 3H), 5.80 (s, 1H), 4.09 (q, *J* = 7.1 Hz, 2H), 2.43 (s, 3H), 1.18 (t, *J* = 7.1 Hz, 3H) ppm.

$^{13}C$ NMR (100 MHz, $CDCl_3$, δ): 166.8, 163.1, 144.8, 144.6, 144.3, 143.0, 128.5, 127.5, 127.4, 123.3, 100.5, 56.0, 59.4, 19.1, 14.4 ppm.

**General method for the synthesis of cationic HAPs 3a-d**

To a stirred solution of HAP derivative **1a** (200 mg, 0.505 mmol, 1.00 eq) in $CCl_4$ (5 mL), NBS (94 mg, 0.530 mmol, 1.05 eq) was added in small portions. The resulting reaction mixture was stirred for 2.5 h at 40°C. Then the solvent was evaporated and purified with flash chromatography on silica gel with EtOAc (5 – 50%) in petroleum ether (bp 40-60°C) to yield methyl 6-(bromomethyl)-4-(2-chloro-4-fluorophenyl)-2-(3,5-difluoropyridin-2-yl)-1,4-dihydropyrimidine-5-carboxylate (**2**) (0.2 g, 80%). $^1H$ and $^{13}C$ NMR spectral data of the compound **2** were in agreement with those reported in literature [3].

To a stirred solution of bromomethyl derivative **2** (0.1 g, 0.205 mmol, 1.00 eq) in dry acetone (2 mL) the corresponding tertiary amine (1.50 eq) was added. The reaction mixture was stirred at room temperature for 4 days after which the resulting precipitate was filtered off and recrystalized from acetone to yield pure products **3a-d.**

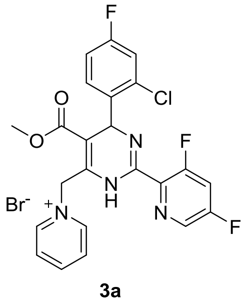

**3a**

**1-[(6-(2-Chloro-4-fluorophenyl)-2-(3,5-difluoropyridin-2-yl)-5-(methoxycarbonyl)-3,6-dihydropyrimidin-4-yl-methyl]-pyridin-1-ium bromide (3a):**

Yield: 40.86 mg, 36%

Melting point (acetone): 173-174°C

Anal. calcd for $C_{23}H_{17}BrClF_3N_4O_2$: C, 49.89; H, 3.09; N, 10.12; found: C, 49.31; H, 3.14; N, 9.89.

$^1H$ NMR (400 MHz, $CDCl_3$, δ): 9.41 (dd, *J* = 6.6, 1.2 Hz, 2H), 8.60 (tt, *J* = 7.8, 1.2 Hz, 1H), 8.23 (d, *J* = 2.3 Hz, 1H), 8.16 (dd, *J* = 6.6, 1.2 Hz, 2H), 8.07 (s, 1H), 7.52 (dd, *J* = 8.6, 5.9 Hz, 1H), 7.20 (ddd, *J* = 10.3, 7.9, 2.3 Hz, 1H), 7.15 – 7.06 (m, 2H), 6.40 (AB-system, 2H, *J* = 15.5 Hz), 6.12 (d, *J* = 2.6 Hz, 1H), 3.69 (s, 3H) ppm.

$^{13}C$ NMR (100 MHz, $CDCl_3$, δ): 165.5, 162.4 (d, *J* = 251.9 Hz), 160.8 (dd, *J* = 269.2, 6.4 Hz), 159.4 (dd, *J* = 279.6, 6.4 Hz), 152.3 (d, *J* = 9.8 Hz), 150. 6, 146.8, 145.6, 135.5 (d, *J* = 3.7 Hz), 133.5 (dd, *J* = 24.3, 4.7 Hz), 132.5 (d, *J* = 10.1 Hz), 132.53 – 132.48 (m), 131.4 (d, *J* = 9.1 Hz), 127.8, 117.5 (d, *J* = 25.0 Hz), 115.7 (d, *J* = 21.1 Hz), 114.2 (t, *J* = 21.9 Hz), 105.2, 63.2, 52.5, 49.6 ppm.

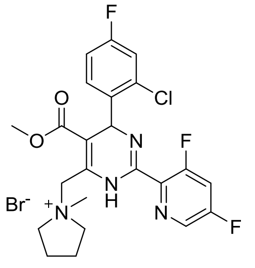


**3b**

**1-[(6-(2-Chloro-4-fluorophenyl)-2-(3,5-difluoropyridin-2-yl)-5-(methoxycarbonyl)-3,6-dihydropyrimidin-4-yl-methyl]-1-methylpyrrolidin-1-ium bromide (3b):**

Yield: 22.95 mg, 20%
Melting point (acetone): 167-168°C
Anal. calcd for $C_{23}H_{23}BrClF_3N_4O_2$: C, 49.35; H, 4.14; N, 10.01; found: C, 48.73; H, 4.20; N, 9.34.
$^{1}H$ NMR (400 MHz, $CDCl_3$, δ): 8.31 (d, *J* = 2.6 Hz, 1H), 8.15 (s, 1H), 7.39 – 7.28 (m, 2H), 7.16 (dd, *J* = 8.3, 2.6 Hz, 1H), 7.03 (ddd, *J* = 8.7, 7.8, 2.6 Hz, 1H), 6.16 (d, *J* = 2.5 Hz, 1H), 4.97 (AB-system, 2H, *J* = 13.8 Hz), 4.22 – 4.09 (m, 4H), 3.64 (s, 3H), 3.55 (s, 3H), 2.47 – 2.25 (m, 4H) ppm.
$^{13}C$ NMR (100 MHz, $CDCl_3$, δ): 165.5, 162.4 (d, *J* = 252.5 Hz), 161.0 (dd, *J* = 269.3, 6.5 Hz), 159.5 (dd, *J* = 280.4, 6.5 Hz), 151.9 (d, *J* = 9.7 Hz), 149.1, 135.6 (d, *J* = 3.5 Hz), 133.8 (dd, *J* = 24.4, 4.6 Hz), 132.7 (d, *J* = 10.4 Hz), 132.5 – 132.4 (m), 130.7 (d, *J* = 9.1 Hz), 117.8 (d, *J* = 24.9 Hz), 115.6 (d, *J* = 21.1 Hz), 114.4 (t, *J* = 22.0 Hz), 110.3, 66.17, 66.13, 62.7, 52.5, 50.2, 49.7, 22.0, 21.9 ppm.

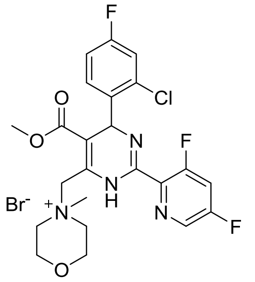


**3c**

**4-[(6-(2-Chloro-4-fluorophenyl)-2-(3,5-difluoropyridin-2-yl)-5-(methoxycarbonyl)-3,6-dihydropyrimidin-4-yl-methyl]-4-methylmorpholin-4-ium bromide (3c):**

Yield: 29.51 mg, 25%
Melting point (acetone): 181-182°C
Anal. calcd for $C_{23}H_{23}BrClF_3N_4O_3$: C, 47.98; H, 9.73; N, 4.03; found: C, 47.54; H, 4.33; N, 9.39.
$^{1}H$ NMR (400 MHz, $CD_3OD$, δ): 8.44 (d, *J* = 2.3 Hz, 1H), 7.74 (ddd, *J* = 10.9, 8.5, 2.3 Hz, 1H), 7.55 (dd, *J* = 8.6, 6.0 Hz, 1H), 7.28 (dd, *J* = 8.6, 2.6 Hz, 1H), 7.12 (td, *J* = 8.6, 2.6 Hz, 1H), 6.23 (s, 1H), 5.19 – 4.81 (AB-system, 2H, *J* = 13.4 Hz), 4.20 – 3.86 (m, 8H), 3.66 (s, 3H), 3.48 (s, 3H) ppm.

$^{13}C$ NMR (100 MHz, $CD_3OD$, δ): 167.0, 163.7 (d, *J* = 250.3 Hz), 162.3 (dd, *J* = 275.4, 6.5 Hz), 161.0 (dd, *J* = 266.5, 6.5 Hz), 153.9 (d, *J* = 8.9 Hz), 148.9, 138.4 (d, *J* = 3.6 Hz), 135.0 (dd, *J* = 24.5, 4.3 Hz), 135.0 – 134.9 (m), 134.1 (d, *J* = 10.6 Hz), 132. 8 (d, *J* = 9.2 Hz), 118.2 (d, *J* = 25.2 Hz), 116.1 (d, *J* = 21.4 Hz), 115.1 (t, *J* = 22.8 Hz), 112.2, 63.3, 63.7, 61.9, 52.5, 51.6 ppm.
$^{19}F$ NMR (376 MHz, $CD_3OD$, δ): -112.6 -112.9 (td, *J* = 8.3, 5.9 Hz), -119.4 (dd, *J* = 11.8, 8.4 Hz) ppm.

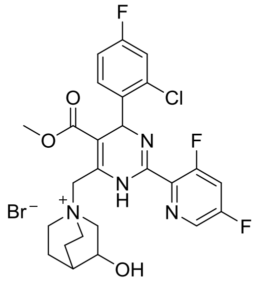


**3d**

**1-[(6-(2-Chloro-4-fluorophenyl)-2-(3,5-difluoropyridin-2-yl)-5-(methoxycarbonyl)-3,6-dihydropyrimidin-4-yl-methy]-3-hydroxyquinuclidin-1-ium bromide (3d):**

Yield: 30.2 mg, 10%
Anal. calcd for $C_{25}H_{25}BrClF_3N_4O_3$: C, 49.89; H, 4.19; N, 9.31; found: C, 49.43; H, 4.60; N, 9.44.
$^{1}H$ NMR (400 MHz, $CD_3OD$, δ): 8.62 (d, *J* = 2.3 Hz, 1H), 8.56 (s, 1H), 7.66 – 7.56 (m, 2H), 7.30 (td, *J* = 8.4, 2.6 Hz, 1H), 7.19 (td, *J* = 8.4, 2.6 Hz, 1H), 4.76 (s, 1H), 4.31 – 4.20 (m, 2H), 4.15 – 3.89 (m, 3H), 3.86 – 3.78 (m, 4H), 3.71 (s, 3H), 2.45 – 2.31 (m, 2H), 2.28 – 2.13 (m, 3H) ppm.
$^{13}C$ NMR (100 MHz, $CD_3OD$, δ): 166.5, 161.9 (dd, *J* = 239.2, 6.7 Hz) 161.4 (d, *J* = 415.2 Hz), 160.8 (dd, *J* = 247.0, 6.7 Hz), 155.1 (d, J = 10.2 Hz),149.0, 135.9 (dd, *J* = 24.1, 4.4 Hz), 135.0 – 134.9 (m), 134.6 (d, *J* = 3.8 Hz), 134.3 (d, *J* = 10.8 Hz), 133.5 (d, *J* = 9.5 Hz), 118.0 (d, *J* = 26.0 Hz), 115.7 (d, *J* = 22.0 Hz), 115.1 (t, *J* = 23.3 Hz), 65.9, 65.54, 65.52, 57.8, 56.2, 54.0, 28.1, 23.8, 22.6 ppm.
$^{19}F$ NMR (376 MHz, $CD_3OD$) δ -110.5 (q, *J* = 7.7 Hz), -115.5 (dd, *J* = 11.6, 8.6 Hz), -119.8 – -120.3 (m) ppm.

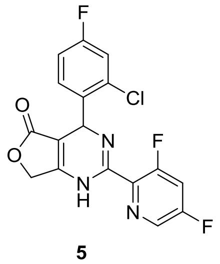


**5**

**4-(2-Chloro-4-fluorophenyl)-2-(3,5-difluoropyridin-2-yl)-4,7-dihydrofuro[3,4-*d*]pyrimidin-5(1*H*)-one (5)**

To a stirred solution of HAP derivative **1a** (250 mg, 0.633 mmol, 1.00 eq) in $CCl_4$ (30 mL), NBS (124 mg, 0.696 mmol, 1.10 eq) was added in small portions. The reaction mixture was stirred for 2.5 h at 40°C and then solvent was evaporated. Crude product was dissolved in $CHCl_3$ (20 mL) and stirred at reflux for 6h. Product was purified by flash chromatography on silica gel with $CH_2Cl_2$/petroleum ether (bp 40-60°C) (2:1) to yield pure product **5** (43 mg, 18%) as yellow powder.
Melting point (methanol): 179-180°C

Anal. calcd for $C_{17}H_9ClF_3N_3O_2$: C, 53.77; H, 2.39; N, 11.07; found: C, 53.69; H, 2.46; N, 10.89.
$^1$H NMR (400 MHz, $CDCl_3$, δ): 8.32 (d, *J* = 2.3 Hz, 1H), 8.08 (s, 1H), 7.37 (ddd, *J* = 10.3, 7.8, 2.3 Hz, 1H), 7.30 (dd, *J* = 8.7, 5.9 Hz, 1H), 7.18 (dd, *J* = 8.3, 2.6 Hz, 1H), 7.02 (ddd, *J* = 8.7, 7.8, 2.6 Hz, 1H), 6.21 (s, 1H), 4.95 (AB-system, 2H, *J* = 16.7 Hz) ppm.
$^{13}$C NMR (100 MHz, $CDCl_3$, δ): 169.5, 162.6 (d, *J* = 252.3 Hz), 160.9 (dd, *J* = 280.2, 6.6 Hz), 159.6 (dd, *J* = 269.6, 6.6 Hz), 154.8 (d, *J* = 9.7 Hz), 134.2 (d, *J* = 3.5 Hz), 133.9 – 133.3 (m), 133.2 – 133.1 (m), 131.2 (d, *J* = 9.2 Hz), 117.8 (d, *J* = 24.9 Hz), 115.1 (d, *J* = 21.3 Hz), 114.45 (t, *J* = 21.8 Hz), 102.2, 70.2, 50.1 ppm.

**General method for aromatization of HAPs 1j,k**

To a stirred solution of HAP derivative **1j** or **1k** (0.60 mmol, 1.00 eq) in toluene (5 mL), 2,3-dichloro-5,6-dicyano-1,4-benzoquinone (DDQ, 163 mg, 0.72 mmol, 1.20 eq) was added, after which the resulting reaction mixture was stirred at 50$^o$C for 2h. After evaporatetion the remaining suspension was diluted with water (5 mL) and extracted with EtOAc (4×10 mL). Organic layers were combined, washed with brine, dried with anhydrous $Na_2SO_4$, filtered and evaporated. The obtained residue was purified with flash chromatography on silica gel with EtOAc (10 – 60%) in petroleum ether (bp 40-60$^o$C)yielding partially purified product. The product was additionally purified by flash chromatography on C18 silica gel with $H_2O$/MeCN to yield pure product **4a** or **4b**.

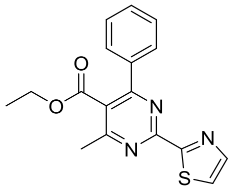


**4a**

**Ethyl 4-methyl-6-phenyl-2-(thiazol-2-yl)pyrimidine-5-carboxylate (4a):**

Yield: 166 mg, 85%
Melting point (MeOH): 93-94$^o$C
Calcd for $C_{17}H_{16}N_3O_2S$ $[M+H]^+$: 326.0885; found: 326.0963.
$^1$H NMR (400 MHz, $CDCl_3$, δ): 8.07 (d, *J* = 3.1 Hz, 1H), 7.77 – 7.73 (m, 2H), 7.56 (d, *J* = 3.1 Hz, 1H), 7.51 – 7.44 (m, 3H), 4.23 (q, *J* = 7.1 Hz, 2H), 2.74 (s, 3H), 1.09 (t, *J* = 7.1 Hz, 3H) ppm.
$^{13}$C NMR (100 MHz, $CDCl_3$, δ): 167.9, 166.7, 166.6, 164.2, 158.7, 145.5, 137.4, 130.5, 128.71, 128.69, 125.2, 123.7, 62.2, 22.9, 13.8 ppm.

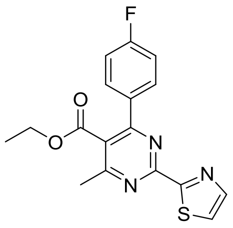

**4b**

**Ethyl 4-(4-fluorophenyl)-6-methyl-2-(thiazol-2-yl)pyrimidine-5-carboxylate (4b):**

Yield: 157 mg, 76%
Melting point (MeOH): 92-93°C
Calcd for $C_{17}H_{15}FN_3O_2S$ $[M+H]^+$: 344.0791; found: 344.0869.
$^1$H NMR (400 MHz, $CDCl_3$, δ): 8.07 (d, *J* = 3.1 Hz, 1H), 7.79 – 7.74 (m, 2H), 7.56 (d, *J* = 3.1 Hz, 1H), 7.20 – 7.13 (m, 2H), 4.25 (q, *J* = 7.1 Hz, 2H), 2.72 (s, 3H), 1.15 (t, *J* = 7.1 Hz, 3H) ppm.
$^{13}$C NMR (100 MHz, $CDCl_3$, δ): 167.8, 166.6, 165.6, 164.9 (d, *J* = 377.1 Hz), 165.6, 158.8, 145.5, 133.5 (d, *J* = 3.3 Hz), 130.9 (d, *J* = 8.5 Hz), 125.0, 123.7, 115.9 (d, *J* = 21.8 Hz), 62.3, 22.9, 13.9 ppm.
$^{19}$F NMR (376 MHz, $CDCl_3$, δ): -109.8 – -109.9 (m) ppm.

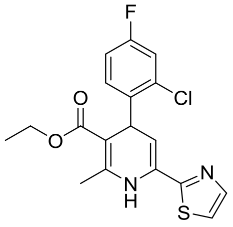

**6**

**Ethyl 4-(2-chloro-4-fluorophenyl)-2-methyl-6-(thiazol-2-yl)-1,4-dihydropyridine-3-carboxylate (6):**

To a solution of 2-chloro-4-fluorobenzaldehyde (1.58 g, 10 mmol) in EtOH (10 mL), a solution of sodium hydroxide in water (10%, 2.5 mL) was added. The mixture was cooled to 0°C and then 2-acetylthiazole was added drop wise with stirring. After stirring for 5 h, the white precipitate was filtered off, washed with cold EtOH and water yielding (*E,Z*)-3-(2-chloro-4-fluorophenyl)-1-(thiazol-2-yl) propenone (2.4 g, 90%) as white powder, which was used in the next step without purification.
The mixture of ethyl-3-aminobut-2-enoate (0.24 g, 1.87 mmol, 1.00 eq) and (*E,Z*)-3-(2-chloro-4-fluorophenyl)-1-(thiazol-2-yl) propenone (0.5 g, 1.87 mmol, 1.00 eq) in ethanol (10 mL) was refluxed for 6h in the presence of ammonium acetate (0.25 g). After cooling, the precipitate was filtered off and crystallized from ethanol, yielding dihydropyridine **6** (0.55 g, 78%) as a light-yellow powder.
Melting point (EtOH): 158-159°C
Anal. calcd for $C_{18}H_{16}ClFN_2O_2S$: C, 57.07; H, 4.26; N, 7.39; found: C, 56.85; H, 4.17; N, 7.09.
$^1$H NMR (400 MHz, $CDCl_3$, δ): 7.70 (d, *J* = 3.2 Hz, 1H), 7.30 (dd, *J* = 8.7, 6.2 Hz, 1H), 7.23 (d, *J* = 3.2 Hz, 1H), 7.07 (dd, *J* = 8.76, 2.6 Hz, 1H), 6.92 (td, *J* = 8.3, 2.6 Hz, 2H), 5.63 (dd, *J* = 5.5, 1.9 Hz, 1H), 5.20 (d, *J* = 5.5 Hz, 1H), 4.03 – 3.93 (m, 2H), 2.51 (s, 3H), 1.05 (t, *J* = 7.1 Hz, 3H) ppm.
$^{13}$C NMR (100 MHz, $CDCl_3$, δ): 167.8, 162.7, 161.0 (d, *J* = 247.8 Hz), 148.2, 142.3, 141.0 (d, *J* = 3.4 Hz), 131.9 (d, *J* = 10.1 Hz), 131.1 (d, *J* = 8.7 Hz), 129.4, 119.1, 116.3 (d, *J* = 24.5 Hz), 114.7 (d, *J* = 20.7 Hz), 106.2, 97.2, 59.5, 37.3, 20.3, 14.2 ppm.

**1-Alkyl-4-methylpyridin-1-ium 4-methylbenzenesulfonates 7a, 7b**

1-Alkyl-4-methylpyridin-1-ium 4-methylbenzenesulfonates **7a** and **7b** were synthesized according to Dubur *et al* [4]. The mixture of 4-picoline (0.93g, 10 mmol) and appropriate hexyl- or hexadecyl 4-methylbenzenesulfonate (10 mmol) in 2-propanol (8 mL) was refluxed for 6 h. The solvent was removed under reduced pressure. The residue was crystallized from ethyl acetate yielding **7a** and **7b** as white powders.

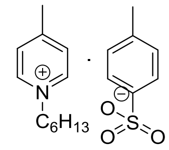


**7a**

**1-Hexyl-4-methylpyridin-1-ium 4-methylbenzenesulfonate (7a)**

Yield: 297 mg, 85%.
Melting point (EtOH): 67 - 69°C
Anal. calcd for $C_{19}H_{27}NO_3S$: C, 65.30; H, 7.79; N, 4.01; found: C, 65.21; H, 7.83; N, 4.20.
$^1$H NMR (400 MHz, $CDCl_3$, δ): 9.10 – 8.98 (m, 2H), 7.90 – 7.60 (m, 4H), 7.23 -6.98 (m, 2H), 4.64 (t, *J*=7.4 Hz, 2H), 2.57 (s, 3H), 2.34 (s, 3H), 1.87 (t, *J*= 7.4 Hz, 2H), 1.32 – 1.09 (m, 6H),0.90 – 0.64 (m, 3H) ppm.
$^{13}$C NMR (100 MHz, $CDCl_3$, δ): 159.29, 144.39, 144.09, 139.13, 128.76, 128.59, 125.94, 61.23, 31.58, 31.12, 26.66, 22.36, 22.04, 21.27, 13.92 ppm.

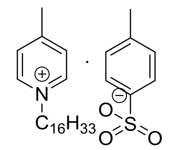


**7b**

**1-Hexadecyl-4-methylpyridin-1-ium 4-methylbenzenesulfonate (7b)**

Yield: 386 mg, 79%.
Melting point (EtOH): 48 - 50°C
Anal. calcd for $C_{29}H_{47}NO_3S$: C, 72.12; H, 9.67; N, 2.86; found: C, 71.98; H, 9.82; N, 2.71.
$^1$H NMR (400 MHz, $CDCl_3$, δ): 8.99 (d, *J*= 6.3 Hz, 2H), 7.72 (dd, *J*= 8.1, 6.3 Hz, 4H), 7.10 (d, *J*= 7.7 Hz, 2H), 4.64 – 4.49 (m, 2H), 2.52 (d, *J*= 3.5 Hz, 3H), 2.31 (s, 3H), 1.82 (t, *J*= 7.2 Hz, 2H), 1.38 – 1.05 (m, 28H), 0.95 – 0.75 (m, 3H) ppm.
$^{13}$C NMR (100 MHz, $CDCl_3$, δ): 159.26, 144.35, 143.92, 139.21, 128.76, 128.62, 128.59, 127.75, 125.93, 61.20, 31.92, 31.62, 29.71, 29.68, 29.66, 29.64, 29.56, 29.41, 29.36, 29.09, 26.02, 22.69, 22.46,22.01, 21.27, 14.12 ppm.

References:


1. Boucle, S.; Lu, X.; Bassit, L.; Ozturk, T.; Ollinger Russell, O.; Amblard, F.; Coats, S.J.; Schinazi, R.F. Synthesis and antiviral evaluation of novel heteroarylpyrimidines analogs as HBV capsid effectors. *Bioorg Med ChemLett* **2017**, *27*, 904-910. Doi: 10.1016/j.bmcl.2017.01.010.
2. Stolting, J.; Stoltefuss, J.; Goldmann, S.; Kramer, T.; Schlemmer, K.H.; Niewohner, U.; Paessens, A.; Graef, E.; Lottmann, S.; Deres, K.; Weber, O. Dihydropyrimidines and their use in the treatment of hepatitis B. Oct. **2000**, Patent WO 00/58302.
3. Mingzhe, J.; Hua B.; Lifei, L.; Yongxiang, G.; Yi, L.; Qixiong, C.; Yongxian, D.; Jian, C.; Hongying, L. Dihydropyrimidines, preparation method and use thereof. March 2018, Patent WO2018045911 A1.
4. Dubur, G.Y.; Dobretsov, G.E.; Deme, A.K.; Dubure, R.R.; Lapshin, E.N.; Spirin, M.M. Fluorescent probes based on styrylpyridinium derivatives: optical properties and membrane binding. *J Biochem Biophys Meth* **1984**, *10*, 123-134.
